# Longitudinal Transcriptomic, Proteomic, and Metabolomic Response of *Citrus sinensis* to *Diaphorina citri* Inoculation of *Candidatus* Liberibacter asiaticus

**DOI:** 10.1021/acs.jproteome.3c00485

**Published:** 2024-02-19

**Authors:** Rachel L. Lombardi, John S. Ramsey, Jaclyn E. Mahoney, Michael J. MacCoss, Michelle L. Heck, Carolyn M. Slupsky

**Affiliations:** † Department of Food Science and Technology, University of California Davis, Davis, California 95616, United States; ‡ Agricultural Research Service, Emerging Pests and Pathogens Research Unit, Ithaca, New York 14853, United States; § Boyce Thompson Institute for Plant Research, Ithaca, New York 14853, United States; ∥ Department of Genome Sciences, University of Washington, Seattle, Washington 98195, United States; ⊥ Plant Pathology and Plant Microbe Biology Section, School of Integrative Plant Science, Cornell University, Ithaca, New York 14853, United States; # Department of Nutrition, University of California Davis, Davis, California 95616, United States

**Keywords:** Huanglongbing, citrus greening disease, systems biology, transcriptomics, proteomics, metabolomics, Asian Citrus Psyllid, ACP, *Diaphorina citri*, *Citrus sinensis*, citrus

## Abstract

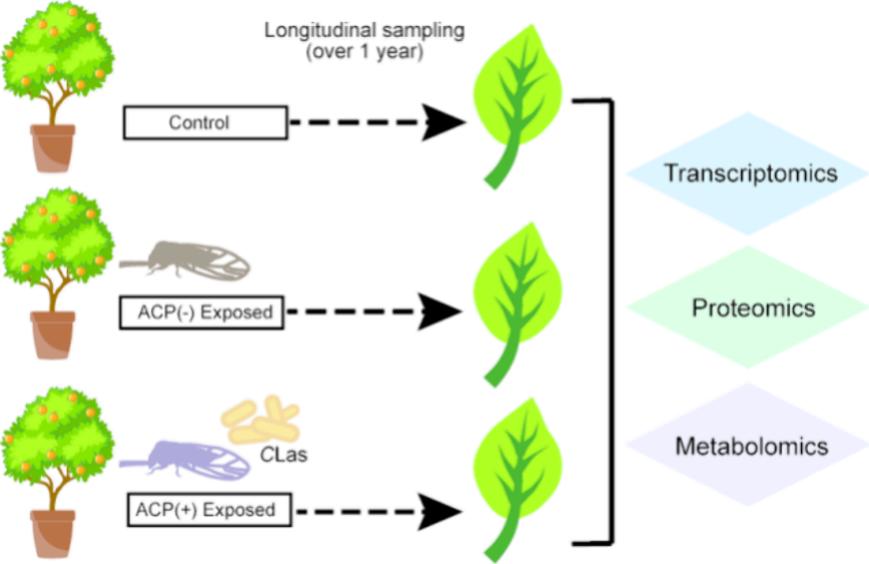

Huanglongbing (HLB) is a fatal citrus disease that is currently threatening citrus varieties worldwide. One putative causative agent, *Candidatus* Liberibacter asiaticus (*C*Las), is vectored by *Diaphorina citri*, known as the Asian citrus psyllid (ACP). Understanding the details of *C*Las infection in HLB disease has been hindered by its *Candidatus* nature and the inability to confidently detect it in diseased trees during the asymptomatic stage. To identify early changes in citrus metabolism in response to inoculation of *C*Las using its natural psyllid vector, leaves from Madam Vinous sweet orange (*Citrus sinensis* (L.) Osbeck) trees were exposed to *C*Las-positive ACP or *C*Las-negative ACP and longitudinally analyzed using transcriptomics (RNA sequencing), proteomics (liquid chromatography-tandem mass spectrometry; data available in Dryad: 10.25338/B83H1Z), and metabolomics (proton nuclear magnetic resonance). At 4 weeks postexposure (wpe) to psyllids, the initial HLB plant response was primarily to the ACP and, to a lesser extent, the presence or absence of *C*Las. Additionally, analysis of 4, 8, 12, and 16 wpe identified 17 genes and one protein as consistently differentially expressed between leaves exposed to *C*Las-positive ACP versus *C*Las-negative ACP. This study informs identification of early detection molecular targets and contributes to a broader understanding of vector-transmitted plant pathogen interactions.

## Introduction

Huanglongbing (HLB) is a fatal citrus disease, currently threatening all commercially relevant citrus varieties worldwide. In the United States, HLB is associated with infection of the fastidious, phloem-restricted α-proteobacterium *Candidatus* Liberibacter asiaticus (*C*Las).^1^*C*Las is transmitted from infected to healthy trees by its psyllid vector *Diaphorina citri* Kuwayama, commonly known as the Asian citrus psyllid (ACP), that primarily feeds on *Citrus* species,^2,3^ and is spread in a circulative, propagative manner linked to the insect’s development.^4^ Infection with *C*Las begins with an asymptomatic period of six months to several years depending on tree age.^1,5^ Initial visual symptoms of infection include yellow shoots, blotchy mottle, and small lopsided fruit; symptoms progress to branch dieback and tree death typically within five-to-six years.^6^ As currently there are no effective treatments, controlling the spread of HLB relies heavily on managing ACP and removing symptomatic trees to limit the transmission of *C*Las.

To date, most efforts to understand the impact of *C*Las on citrus have been made through graft-inoculation studies. Graft-inoculation has the advantage of simplifying the system to isolate the plant’s response to the pathogen alone; however, it does not capture the plant’s response to ACP feeding or the response to both the pathogen and ACP feeding. Indeed, ACP herbivory has been shown to cause changes in leaf primary and secondary metabolism^7−9^ that can lead to long-term damage in the plant.^2^ Interestingly, there is evidence to suggest that progression of HLB symptoms in citrus may differ when *C*Las is introduced by graft- versus ACP-inoculation.^10^ Indeed, ACP are mobile, and thus can introduce the pathogen throughout the tree canopy while simultaneously inflicting cellular damage to leaves inducing herbivore-associated responses and changes in metabolism.^7−9,11,12^

Studies on the vector-pathogen relationship of ACP and *C*Las have shown that *C*Las induces physiologic, metabolic, and behavioral changes in ACP.^13−18^ However, it is not known whether the impact of *C*Las colonized ACP feeding causes a plant response different from that of just ACP feeding alone. Prior studies have shown that graft-inoculation of *C*Las into sweet orange (*Citrus x sinensis* (L.) Osbeck) results in a limited plant response during the initial phases of the infection.^19,20^ Whether this plant response is conserved during herbivory by ACP colonized with *C*Las remains to be determined. This study is the first year-long longitudinal analysis utilizing transcriptomics, proteomics, and metabolomics to investigate the response of citrus to *C*Las in the context of ACP herbivory.

## Materials and Methods

### Experimental Design

Research involving plants and insects exposed to the plant pathogen *Candidatus* Liberibacter asiaticus (*C*Las) was conducted in accordance with state and federal guidelines and with all necessary permits. A total of 36 Madam Vinous sweet orange (*Citrus sinensis* (L.) Osbeck) trees grown from certified pathogen-free seeds (USDA-ARS National Clonal Germplasm Repository for Citrus & Dates, Riverside, CA) were used. Throughout the experiment, plants were kept in 1-gallon pots in Cornell Soilless Potting Mix, watered three times a week or as needed, and fertilized regularly with Jack’s Professional LX 21-5-20 fertilizer (cat#: 77990) supplemented with 300 ppm of Epsom salt.

Approximately 2.5 weeks before the start of the experiment, all trees were pruned, randomly paired, placed into square 60 cm × 60 cm × 60 cm shared bug dorms, and relocated to one of two insectary chambers, where they were allowed to produce flush. One chamber contained 12 trees to be exposed to *C*Las-negative ACP (hereafter referred to as *C*Las(−) ACP) and 6 control trees that would not be exposed to ACP. The second chamber contained 12 trees to be exposed to *C*Las-positive ACP (hereafter referred to as *C*Las(+) ACP) and 6 control trees. Both insectary chambers were maintained between 24 and 28 °C with a 14 h light:10 h dark photoperiod using high output fluorescent lighting and were not controlled for humidity. In accordance with the USDA-APHIS protocol, trees remained in these chambers from the time of initial ACP exposure until all psyllids were removed via vacuum aspiration (∼21 weeks post exposure (wpe)). At 23 wpe, trees from both insectary chambers were relocated to an insect-free greenhouse, and bug dorms were removed. The greenhouse was maintained at a minimum temperature of 21 °C with supplementary high output fluorescence lighting using a 14 h light:10 h dark photoperiod. Humidity was not controlled. All trees were sprayed with Avid and horticultural oil on an as-needed basis to control spider mites.

Lab propagated colonies of *C*Las(+) ACP and *C*Las(−) ACP reared on *C*Las-positive *Citrus medica* and *C*Las-negative *Citrus macrophylla*, respectively, were acquired from the University of Florida. The *C*Las strain used in this study originated from an HLB diseased tree in South Miami-Dade County, Florida. Both colonies were acclimated for 4 days on flushing *C*Las-negative *Citrus sinensis* (L.) Osbeck prior to the start of the experiment. Detection of *C*Las in the *C*Las(+) ACP colony was carried out using quantitative polymerase chain reaction (qPCR) as described by Hall and Moulton^21^ with no modifications. In brief, DNA was extracted from individual *C*Las(+) ACP which was used as input for qPCR using the HLBaspr primer/probe set designed to target the 16S region of the pathogen.^22^ qPCR of DNA from individual ACP was run in a minimum of two technical replicates with positive and negative controls. Individual insects were considered “positive” for *C*Las when the average qPCR cycle threshold (Ct) value was Ct ≤ 38.^21,23^

Leaf sampling for metabolomics, transcriptomics, proteomics, and qPCR took place from September 2015 to September 2016. Each collection of citrus leaves consisted of four nonflush leaves for metabolomics and transcriptomics and three-to-six nonflush leaves for proteomics and qPCR. For leaves of trees exposed to ACP, honeydew residue was gently removed using a spatula prior to collection. Leaves were clipped from trees, placed into aluminum foil packets, flash frozen in liquid nitrogen, and stored at −80 °C for downstream analysis. Details on the preparation of leaf materials for analysis are provided in Supplementary Methods.

**Figure 1:**
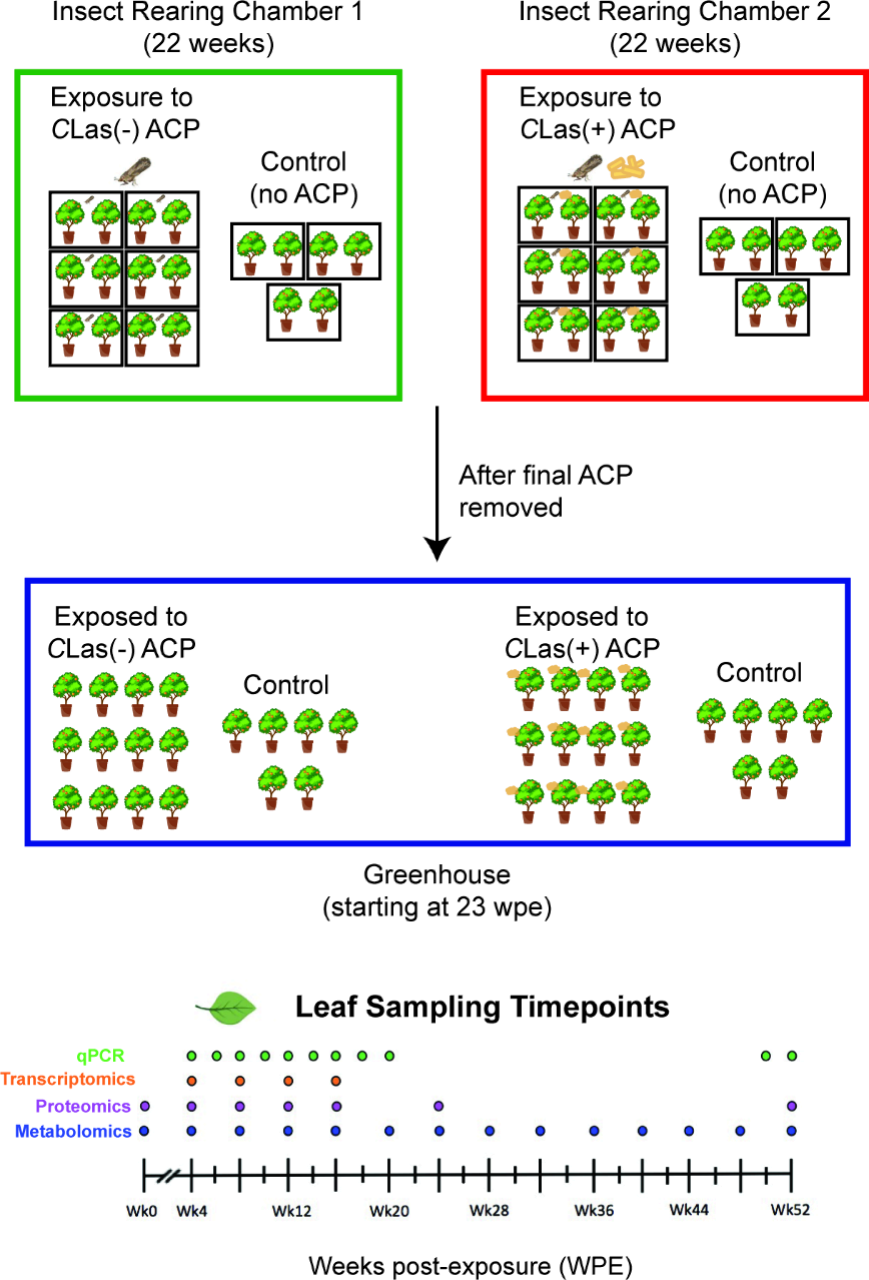
Study design. A total of 36 *Citrus sinensis* (L.) Osbeck trees were housed in pairs in bug dorms, in one of two insectory chambers. In one insectory chamber, 12 of the trees were exposed to *C*Las-free ACP (200 *C*Las(−) ACP were released into each of the bug dorms), and 6 trees were not exposed. In the other chamber, 12 trees were exposed to ACP carrying *C*Las (200 *C*Las(+) ACP were released into each of the bug dorms), and another 6 trees were not exposed. Starting at 2 weeks, adult ACP were removed using vacuum aspiration until the last one was removed at 21 weeks. At 23 weeks, all trees were removed from the chambers and bug dorms and plants were placed into a greenhouse. Leaf sampling occurred throughout and is indicated for qPCR, transcriptomic, proteomic, and metabolomic analyses. ACP: Asian citrus psyllid (insect vector). *C*Las: *Candidatus* Liberibacter asiaticus (bacterial pathogen).

One day after leaf collection for baseline analysis (0 wpe), groups of 200 *C*Las(+) ACP or 200 *C*Las(−) ACP were transferred to the designated bug dorms. Manual vacuum aspiration to remove adult psyllids began at 2 wpe and was repeated once or twice a day over the next 21 weeks as eggs and nymphs progressed into adulthood. Regular leaf sample harvesting began at 4 wpe and continued until 52 wpe. Analyzed time points are provided in Figure 1.

### Confirmation of CLas Infection in Citrus

Because of the small canopy size of young trees, only one leaf was collected every other week from 4 to 22 wpe for qPCR; once a larger canopy size was established, four leaves were collected every other week for the remainder of the experiment (24 wpe to 52 wpe). Leaf petioles were isolated and, when possible, pooled. DNA was extracted from 200 mg (fresh weight) of petiole material using a Qiagen MagAttract plant DNA extraction kit (Qiagen, Valencia, CA). Quantitative PCR (qPCR)^22^ was performed using the USDA-APHIS-PPQ protocol at 4, 6, 50, and 52 wpe for nonexposed and *C*Las(−) ACP exposed trees, and qPCR for *C*Las(+) ACP exposed trees was carried out at 4, 6, 8, 12, 16, 20, 50, and 52 wpe. In accordance with the USDA-APHIS-PPQ standard, trees were considered “positive” for the presence of *C*Las if Ct values were Ct ≤ 36 at more than one time point (USDA-APHIS-PPQ 2012). Technical replicates were used to confirm “positive” Ct values.

### Transcriptomics

A subset of 15 trees was randomly selected to undergo RNA sequencing at four time points (4, 8, 12, and 16 wpe) (Table S1). Detailed information regarding RNA extraction, library preparation, and paired end RNA sequencing can be found in Supplementary Methods.

Raw reads were quality-checked using FastQC 0.11.5^24^ and MultiQC 1.2.^25^ Trimmomatic 0.36^26^ was used to remove potential adapter contamination and low quality base pairs using the following parameters: leading = 2, trailing = 2, slidingwindow = 4:2, minlen = 36. STAR 2.5.2b^27^ was used to align trimmed reads to the *Citrus sinensis* v2.0 HZAU genome^28^ and to generate gene counts using the default parameters.

At each time point, differentially expressed genes were identified in RStudio (v1.1.463)^29^ using the edgeR package (v3.30.3).^30^ Raw gene counts were filtered to exclude genes with less than 1 count per million (CPM) in 5 samples at each time point. Trimmed mean of *M*-values (TMM) normalization was applied before pairwise testing between groups of age-matched trees using quasi-likelihood F-testing (edgeR::glmQLFTest). Genes were considered differentially expressed if the absolute value of the log_2_ fold change (FC) was greater or equal to 1 (|log_2_ FC| ≥ 1) and the Benjamini-Hochberg false discovery rate (FDR) adjusted *p*-value ≤ 0.05.

Pathway enrichment analysis was carried out using CitrusCyc Pathway v4.0 Database.^28,31−33^ Fisher’s exact test (*p* < 0.05, no FDR adjustment) was used to identify enriched pathways. Figures summarizing the top five pathways with the lowest *p*-values for each time point were created in RStudio using the ggplot2 package (v3.3.2).^29^

### Proteomics

Proteomics was carried out on the same subset of trees that underwent RNA sequencing (Table S1). Details on the preparation of leaf samples for protein extraction are described in Supplementary Methods.

MS/MS Thermo *.raw files were converted to Mascot Generic Format (*.mgf) using msConvertGUI (64 bit; ProteoWizard). Mascot Daemon (Matrix Science, London, UK; v2.5.1) was used to search all *.mgf files against a *Citrus sinensis* protein database containing amino acid sequences corresponding to gene coding sequences from the *Citrus sinensis* v2.0 HZAU genome^28^ and common contaminant proteins. Digestion by trypsin was specified. FDR was estimated by searching against a decoy database containing the reverse sequences of all proteins. The search used a fragment ion mass tolerance of 0.60 Da, a parent ion tolerance of 20 PPM, peptide charges of 2+, 3+, and 4+, and a maximum missed cleavage of one. Carbamidomethyl cysteine was included as a fixed modification. Variable modifications included deamidated asparagine and glutamine and the oxidation of methionine.

Scaffold Q+ (v4.11.1, Proteome Software Inc., Portland, OR) was used to generate a list of weighted spectral counts using Cluster Mode. Peptide and protein identification thresholds were set to 95% and a minimum of 2 peptides were required for protein identification. A Fisher exact test (FDR < 0.05; Benjamini–Hochberg correction) was used to identify differentially abundant proteins (DAPs) between experimental groups. CitrusCyc was used to assign one or more root pathway ontologies to each protein when possible and summarized into a figure using the ggplot2 package. Top reoccurring proteins for a given pairwise comparison were identified by consolidating the lists of differentially abundant proteins across time and isolating the most frequently occurring protein names.

Testing for proteome differences was carried out in RStudio using weighted spectral counts for proteins with at least one count as input for permutational multivariate analysis of variance (PERMANOVA) using Euclidian distance (vegan::adonis2, v 2.5–6) followed by subsequent pairwise testing (pairwiseAdonis::adonis, v 0.0.1) (FDR ≤ 0.05).

Principal component analysis (PCA) was completed using weighted spectral counts for proteins with at least one count as input (stats v4.0.1, factoextra v1.0.7). Loading plots were generated, showing only the top 20 variables contributing to principal component 1 (PC1) and principal component 2 (PC2) (ggplot2).

### Metabolomics

Metabolomics was carried out for all trees at 0, 4, 8, 12, 16, 24, 28, 32, 34, 38, 42, 44, 48, and 52 wpe. Proton nuclear magnetic resonance (^1^H NMR) sample preparation and data acquisition were performed as described by Chin et al.^7,34^ with slight modifications. Metabolomics sample preparation and proton NMR data acquisition are described in the Supplementary Methods.

Statistical analyses were conducted in RStudio. Using raw and transformed concentrations as input, a Shapiro–Wilk test was used to test for normality of residuals (stats v4.0.1), a Levene’s test was used to test for heteroskedasticity (car v3.0–9), and boxplots were created to provide a visual summary of the data. Because the assumptions of parametric testing were not met by using raw or transformed data, nonparametric testing was carried out. Data were log_10_ transformed prior to multivariate analysis to control for violations of multivariate homogeneity of groups dispersions (an assumption of PERMANOVA) and prior to univariate analysis to address variation in group distributions, an assumption of Kruskal–Wallis, to allow hypothesis testing based on differences in medians (and not mean-ranks). Testing for differences in the metabolome was carried out using PERMANOVA with Euclidian distances followed by subsequent pairwise testing (FDR ≤ 0.05). Kruskal–Wallis with posthoc Dunn’s multiple comparison test was performed to identify statistically different metabolite concentrations between experimental groups (FDR ≤ 0.05) of age matched trees. Visualization of log_10_ transformed data was carried out using PCA. Loading plots were generated showing only the top 20 variables contributing to PC1 and PC2.

## Results

All trees exposed to *C*Las(+) ACP tested positive for *C*Las (Ct ≤ 36) at least twice on or before 20 wpe (Table S1). Although two trees in the *C*Las (−) ACP exposed group and three trees in the nonexposed control group were found to have Ct ≤ 36 at one time point each, none of these trees demonstrated a *C*Las-positive Ct value otherwise. As foliar symptoms of HLB can develop as early as 6 months postinfection in young *C. sinensis* trees,^5,8,35^ weeks 4, 8, 12, and 16 of this study were identified as time points that would fall within the asymptomatic period.

### Impact of ACP ± CLas Feeding on the Plant Transcriptome

**Figure 2:**
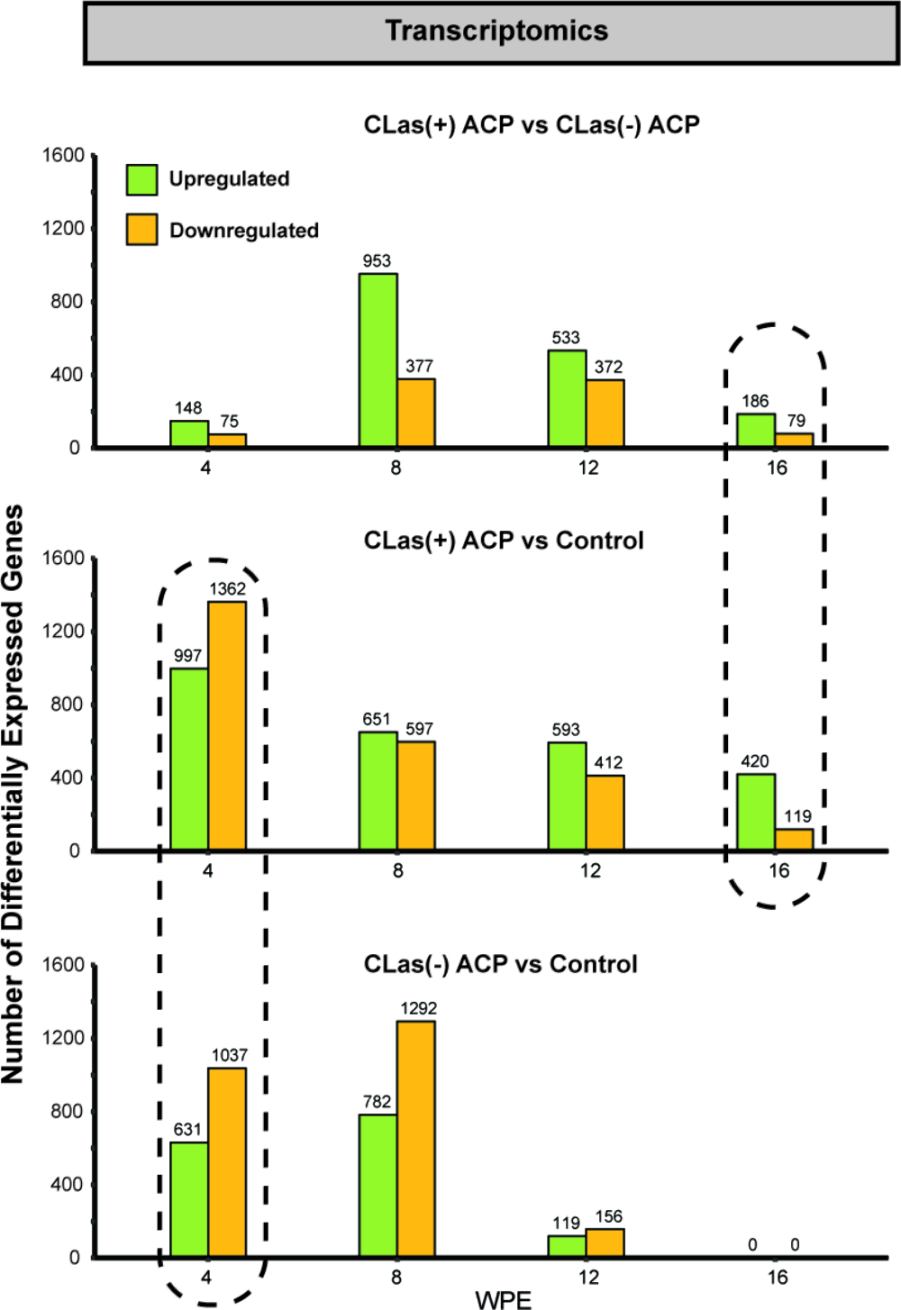
Summary of global transcriptome changes at 4, 8, 12, and 16 wpe expressed as the number of DEG genes between pairwise comparisons of trees exposed to *C*Las(+) ACP, *C*Las(−) ACP, and no ACP (“control”). Comparisons where percent overlap was investigated are outlined with dotted lines.

The average percent of reads mapping to the *Citrus sinensis* genome for each experimental group ranged between 87% and 90% (Table S2), and the number of genes retained after CPM filtering was between 17,070 and 17,524 at each time point. The complexity and high variability of the transcriptome are exemplified when comparing changes in leaf gene expression in response to each stimulus over time. Thus, to disentangle the impact of ACP feeding from the impact of *C*Las on plant response at each time point, three pairwise comparisons were performed: (1) plants exposed to *C*Las(−) ACP vs control; (2) plants exposed to *C*Las(+) ACP vs control; and (3) plants exposed to *C*Las(+) ACP vs *C*Las(−) ACP (Figure 2). Compared to control, a total of 1,668 DEGs were observed in citrus with *C*Las(−) ACP feeding, and 2,359 DEGs were observed with *C*Las(+) ACP feeding, whereas the number of DEGs when comparing citrus with *C*Las(−) ACP and *C*Las(+) ACP feeding was 223. This suggests that ACP feeding was the largest stressor at this time point. Interestingly, *C*Las infection was clearly discernible in the transcriptome at 8 wpe, as a significant number of DEGs (1,330) were observed when comparing *C*Las(+) and *C*Las(−) ACP exposed trees. At 16 wpe, the leaf transcriptome of trees exposed to *C*Las(−) ACP was indistinguishable from control trees, suggesting that as the insect stress is removed, there are no lasting impacts on the leaf transcriptome. For trees exposed to *C*Las(+) ACP relative to control trees, 165 genes were consistently differentially expressed at all four time points; however, only 17 genes were consistently differentially expressed when comparing trees exposed to *C*Las(+) ACP and those exposed to *C*Las(−) ACP (Table 1). Interestingly, these genes maintained consistent up- or downregulation across time.

**Table 1:** Log Fold Change of Genes Consistently and Significantly Differentially Expressed^a^ between Leaves of Trees Exposed to *C*Las(+) ACP or *C*Las(−) ACP at 4, 8, 12, and 16 wpe

Gene	4 WPE	8 WPE	12 WPE	16 WPE	BLAST
Cs2g17510	1.62	1.66	1.60	1.11	Adagio protein 3
Cs4g19660	1.66	1.74	2.14	1.35	Protein NRT1/PTR FAMILY 1.2
Cs5g10870	1.08	1.91	1.69	1.04	NAC domain-containing protein 100
Cs5g25880	1.41	3.12	2.04	1.37	Cytochrome P450 83B1
Cs5g25920	1.19	1.44	1.62	1.09	Cytochrome P450
Cs5g25930	1.37	1.87	2.11	1.16	Cytochrome P450 71A1
Cs7g03390	2.75	2.55	1.77	1.47	–
Cs7g13810	2.02	1.85	2.31	1.30	Chaperone protein dnaJ C76, chloroplastic
Cs7g14760	1.42	1.04	1.90	1.16	Phosphoinositide phospholipase C 2
orange1.1t03148	2.07	1.71	1.89	1.20	Lysine-specific demethylase JMJ30
orange1.1t05692	2.14	1.87	1.91	1.24	Chaperone protein dnaJ C76, chloroplastic
Cs2g04720	–1.17	–1.27	–1.21	–1.45	CBL-interacting protein kinase 5
Cs6g14540	–2.35	–3.40	–2.51	–2.93	Alpha carbonic anhydrase 1, chloroplastic
Cs6g16000	–1.65	–1.99	–1.98	–1.28	Protein REVEILLE 1
Cs7g17100	–1.41	–1.54	–1.24	–1.34	–
Cs8g20490	–1.22	–2.01	–1.37	–1.15	Probable N-acetyltransferase HLS1-like
Cs9g17610	–2.51	–1.84	–1.42	–1.31	–

a All false discovery rate *p*-values from Fisher’s exact test < 0.05.

**Figure 3:**
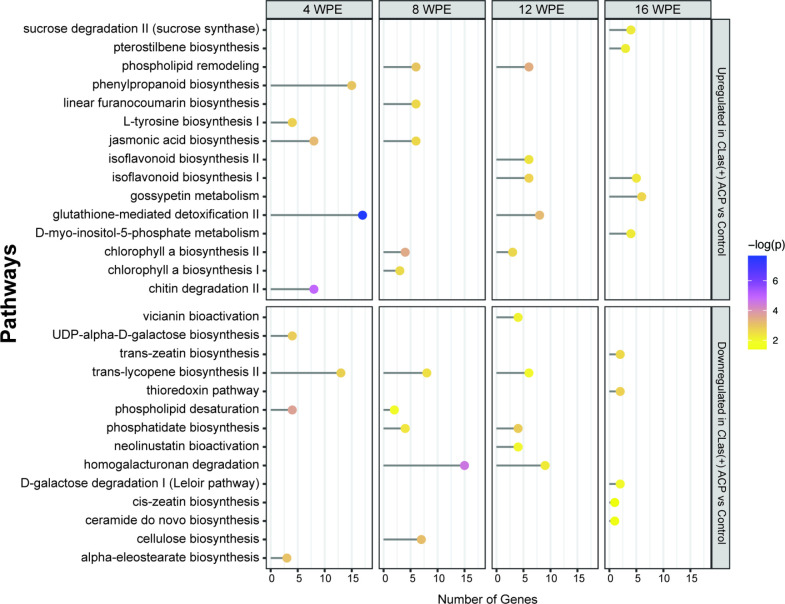
Top five enriched pathways at each time point identified from lists of up- and downregulated genes between trees exposed to *C*Las(+) ACP compared to control trees. The corresponding number of genes assigned to each pathway is also shown.

**Figure 4:**
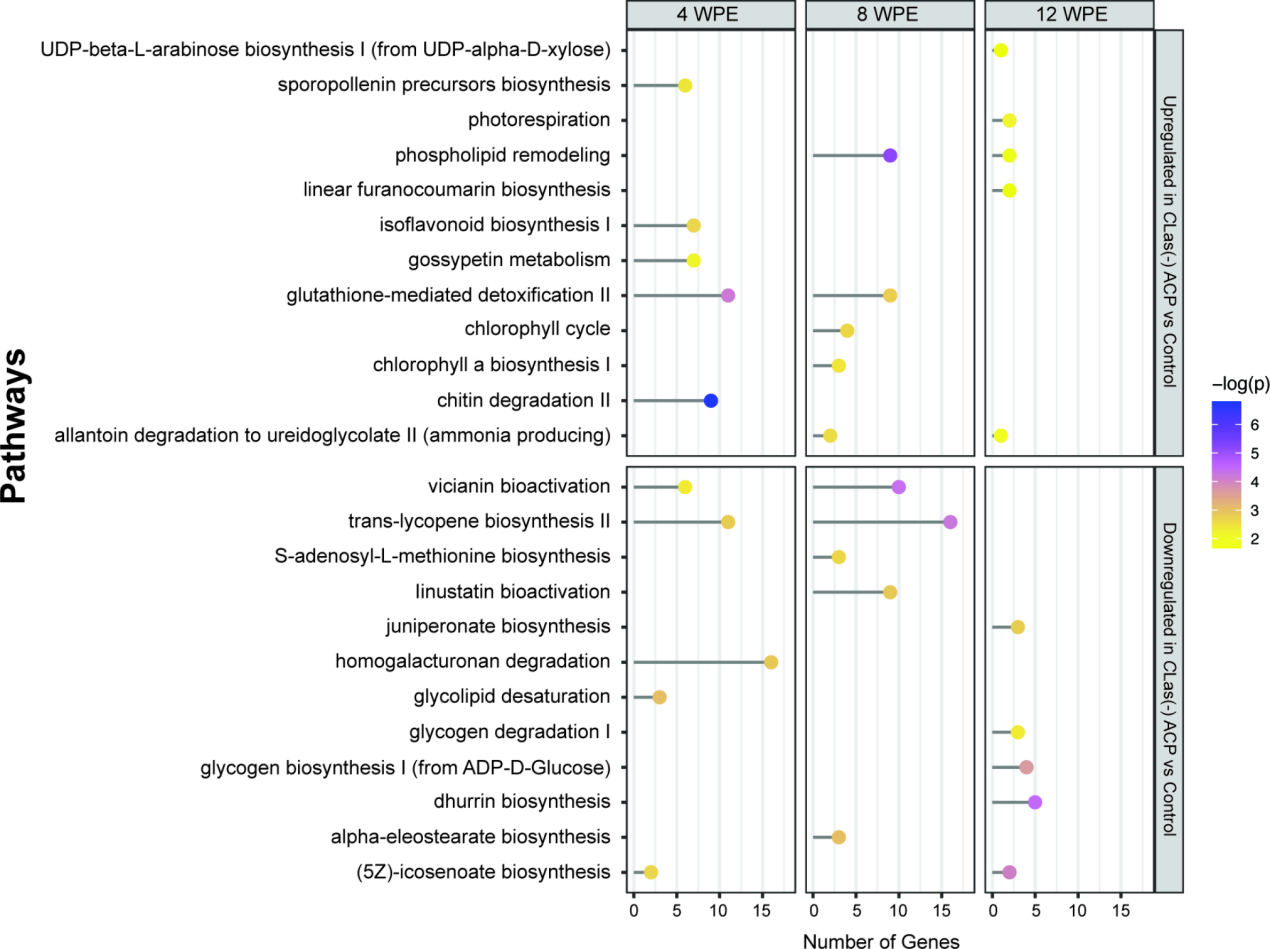
Top five enriched pathways at each time point identified from lists of up- and downregulated genes between trees exposed to *C*Las(−) ACP compared to control trees. The corresponding number of genes assigned to each pathway is shown. No differentially expressed genes were identified between *C*Las(−) ACP and control trees at 16 wpe; therefore, pathway enrichment analysis was not carried out at this time point.

Comparison of metabolic pathways impacted by ACP feeding regardless of whether ACP was carrying *C*Las revealed a down-regulation of genes associated with α-eleostearate biosynthesis at 4 wpe, and up-regulation of phospholipid remodeling at 8 and 12 wpe (Figures 3 and Figure 4). Other pathways, not necessarily up- or downregulated at the same time points, included the phenylpropanoid pathway (linear furanocoumarin synthesis, isoflavonoid biosynthesis I, gossypetin metabolism), carbohydrate degradation (chitin degradation II, homogalacturan degradation), and secondary metabolite metabolism (vicianin bioactivation, trans-lycopene biosynthesis II).

**Figure 5:**
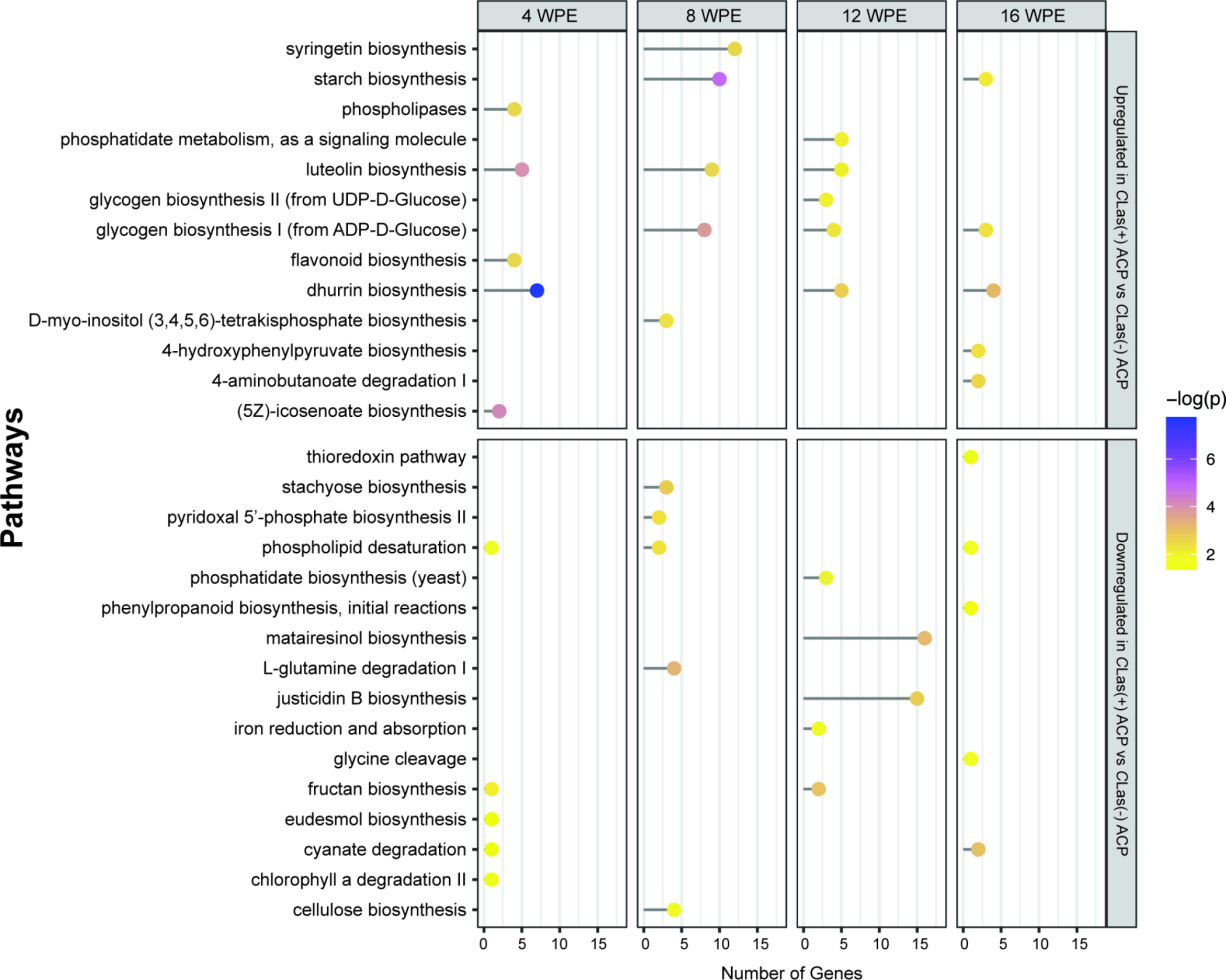
Top five enriched pathways at each time point identified from lists of up- and downregulated genes between trees exposed to *C*Las(+) ACP compared to *C*Las(−) ACP. The corresponding number of genes assigned to each pathway is also shown.

Comparison of the transcriptome of plants exposed to *C*Las(−) ACP and *C*Las(+) ACP feeding at 16 wpe, when the impact of exposure to ACP on the transcriptome was minimal and metabolic pathways are primarily impacted by *C*Las, revealed several pathways that were perturbed at early time points (Figure 5). This included starch biosynthesis, which was upregulated at both 8 and 16 wpe, glycogen biosynthesis I, which was upregulated at 8, 12, and 16 wpe, and cyanogenic glucoside metabolism (dhurrin biosynthesis, which was upregulated at 4, 12, and 16 wpe, and cyanate degradation, which was upregulated at 4 and 16 wpe) in plants exposed to *C*Las(+) ACP compared to plants exposed to *C*Las(−) ACP.

### Impact of ACP ± CLas Feeding on the Plant Proteome

Consistent with the complexity of the transcriptomic results, the proteomic results similarly demonstrated variability in the leaf response over time. Although pairwise testing using PERMANOVA identified no significant differences in the proteome overall, principal component analysis (PCA) at each time point revealed some separation starting at 8 wpe (Figure S1).

**Figure 6:**
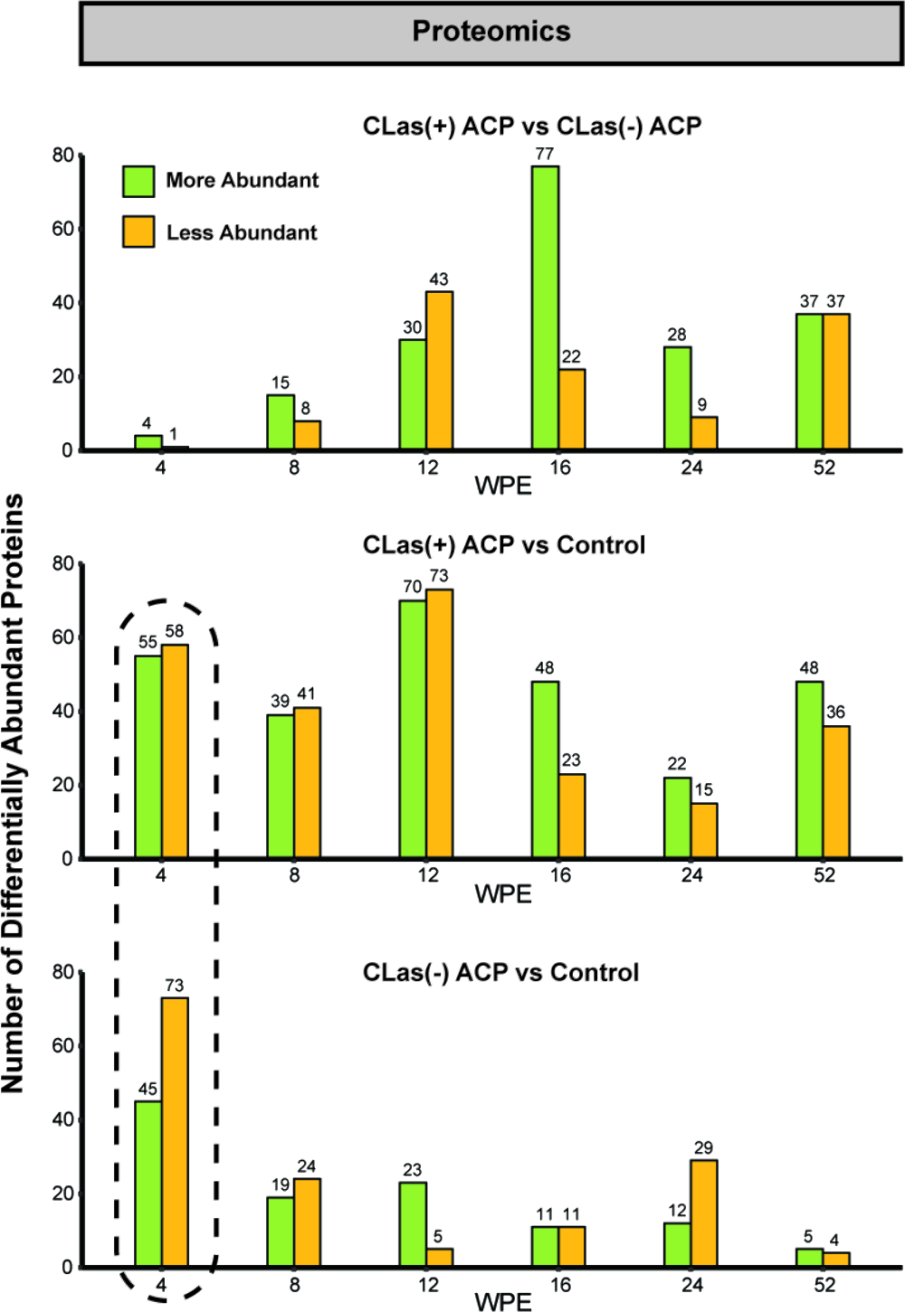
Summary of the global proteome changes between pairwise comparisons of trees exposed to *C*Las(+) ACP, *C*Las(−) ACP, and no ACP (“control”). The numbers of differentially abundant proteins at 4, 8, 12, 16, 24, and 52 wpe are shown. The comparison where percent overlap was investigated is outlined with dotted lines.

**Figure 7:**
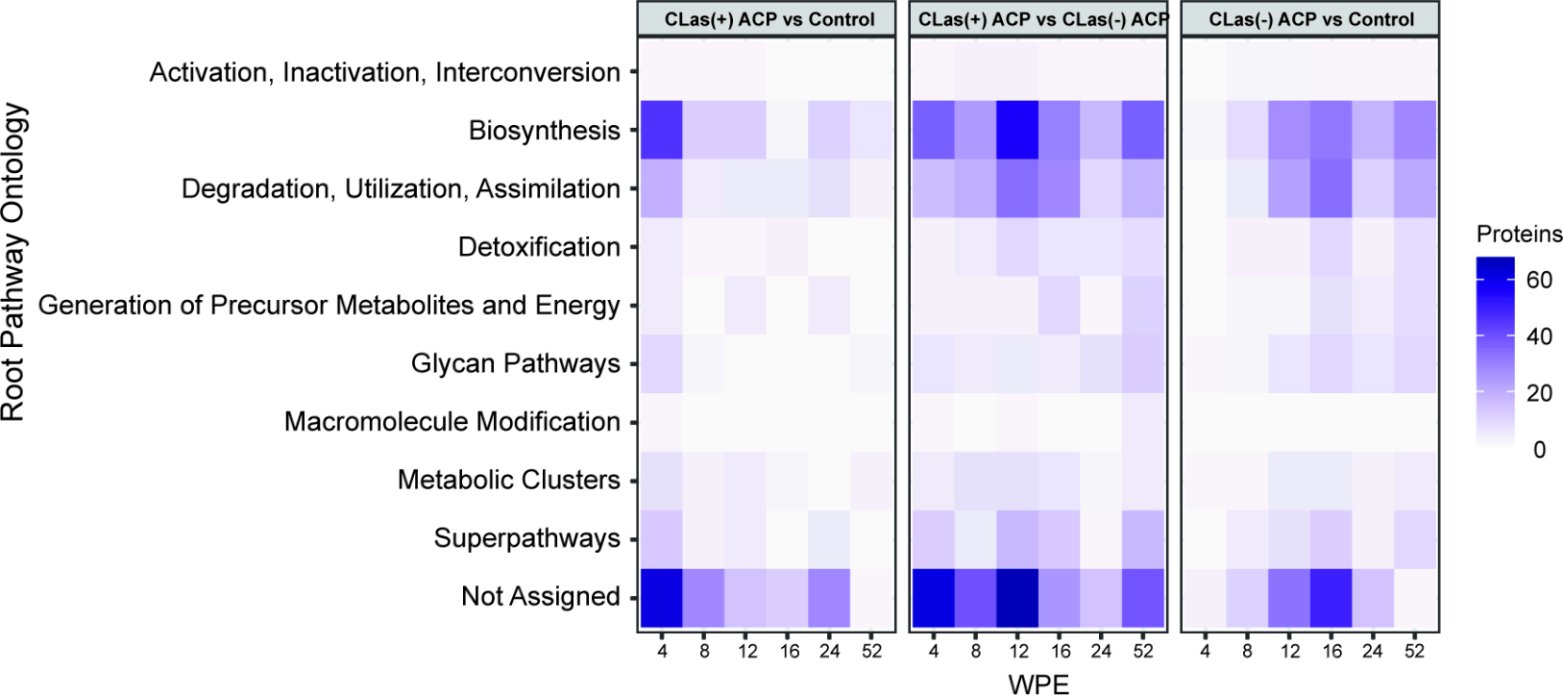
Root pathway ontologies assigned to the differentially abundant proteins between the three pairwise comparisons at 4, 8, 12, 16, 24, and 52 wpe.

The results of Fisher’s exact test for 4 to 52 wpe are summarized in Figure 6. The total number of DAPs identified ranged from 5 to 143 proteins. The largest discrepancy in total protein count was observed at 4 wpe. At this time point, the number of DAPs identified between control trees and those exposed to *C*Las(+) ACP or *C*Las(−) ACP was considerably higher relative to the number identified between *C*Las(+) ACP and *C*Las(−) ACP exposed trees, suggesting that the major stressor at this early time point was ACP feeding, as was observed in the transcriptome data. At week 12 and until week 52, a significant number of proteins were differentially abundant between *C*Las(+) ACP and *C*Las(−) ACP exposed trees. Figure 7 illustrates the root pathway ontologies associated with these DAPs over time, which showed altered distribution of cellular resources pertaining to biosynthesis, degradation/utilization/assimilation, and several proteins that cannot be assigned to any specific pathway or any root pathway ontology, such as membrane bound proteins and those involved in DNA binding.

HLB is associated with alteration of sugar and starch networks within leaves, causing disruption of source-sink relationships. Infection with *C*Las has been shown to result in significant changes to starch metabolism, including accumulation of starch synthase.^36,37^ Notably, we observed the granule-bound starch synthase Ib precursor to be higher in *C*Las(+) ACP trees compared to control and *C*Las(+) ACP compared to *C*Las(−) ACP trees at 8, 12, 16, 24, and 52 wpe. Interestingly, the average fold change of starch synthase increased between the two experimental groups with each successive analysis point throughout the year (Table S3). To a lesser extent, sucrose metabolism has also been shown to be impacted during infection and is associated with repression of sucrose synthase,^36^ though our results suggest that regulation of sucrose synthase may be variable with time, as putative uncharacterized sucrose synthase PtrSuSY1 was lower at 12 wpe, and higher at 16 wpe in *C*Las(+) ACP trees compared to control and *C*Las(+) ACP compared to *C*Las(−) ACP trees.

In addition to changes in sugar networks, HLB is also associated with decreased photosynthesis, and we observed a subtle decrease in chloroplast carbonic anhydrase isoforms in infected leaves starting at 16 wpe, which continued to the end of the study when comparing *C*Las(+) ACP trees to *C*Las(−) ACP trees. The putative chloroplast nucleoid DNA binding protein was also observed to be higher in *C*Las(+) ACP trees compared to the control at 4, 8, 12, 16, 24, and 52 wpe.

The 21 kDa seed protein, a protein with homology to the Kunitz-type inhibitor family of protease inhibitors, and part of a family of inhibitors that has previously been associated with *C*Las infection,^34,38^ was higher in *C*Las(+) ACP trees compared to control at 4, 8, 12, and 16 wpe. Xylem cysteine proteinase 1 was also higher in *C*Las(+) ACP trees at 4, 8, and 12 wpe compared to control. Interestingly, the *C*Las genome codes for sec-delivered effector 1 that has been shown to directly interact with xylem cysteine proteinase 1 and is capable of inhibiting the activity of papain-like cysteine proteases (PLCPs).^39^ PLCPs have been shown to play a role in plant defense during bacterial infection^40^ and in response to herbivory.^41−43^ Chloroplastic linoleate 13S-lipoxygenase 2-1 plays an important role in jasmonic acid biosynthesis, and this protein’s abundance was observed to oscillate throughout the study, starting out higher in trees exposed to *C*Las(+) ACP at 4 and 8 wpe, decreasing in these trees at 12 and 24 wpe, and increasing again by 52 wpe. The THO complex is a conserved nuclear structure involved in the formation of export-competent messenger ribonucleoprotein (mRNP) and plays a pivotal role at the interface between transcription and RNA export. While the exact role of the THO complex and its associated proteins in plants is still evolving, studies in *Arabidopsis thaliana* have demonstrated the complex’s role in siRNA production,^44^ microRNA production,^45^ as well as disease resistance and senescence.^46^ THO complex subunit 4 was found to be higher in *C*Las(+) ACP trees at 16 wpe and lower at 52 wpe.

### Impact of ACP ± CLas Feeding on the Plant Metabolome

Using ^1^H NMR spectroscopy, a total of 27 metabolites were identified and quantified in each sample. Identified metabolites included amino acids, sugars, energy metabolism, and defense-related compounds. As with the transcriptomics and proteomics data, there was substantial variability over time. Trends were apparent with some metabolites, such as aspartate, which demonstrated a gradual decrease in concentration for all trees over time (Figure S2); however, most metabolites exhibited temporal complexity in their abundance likely due to subtle environmental changes, despite efforts to ensure the environment was similar for all plants.

**Figure 8:**
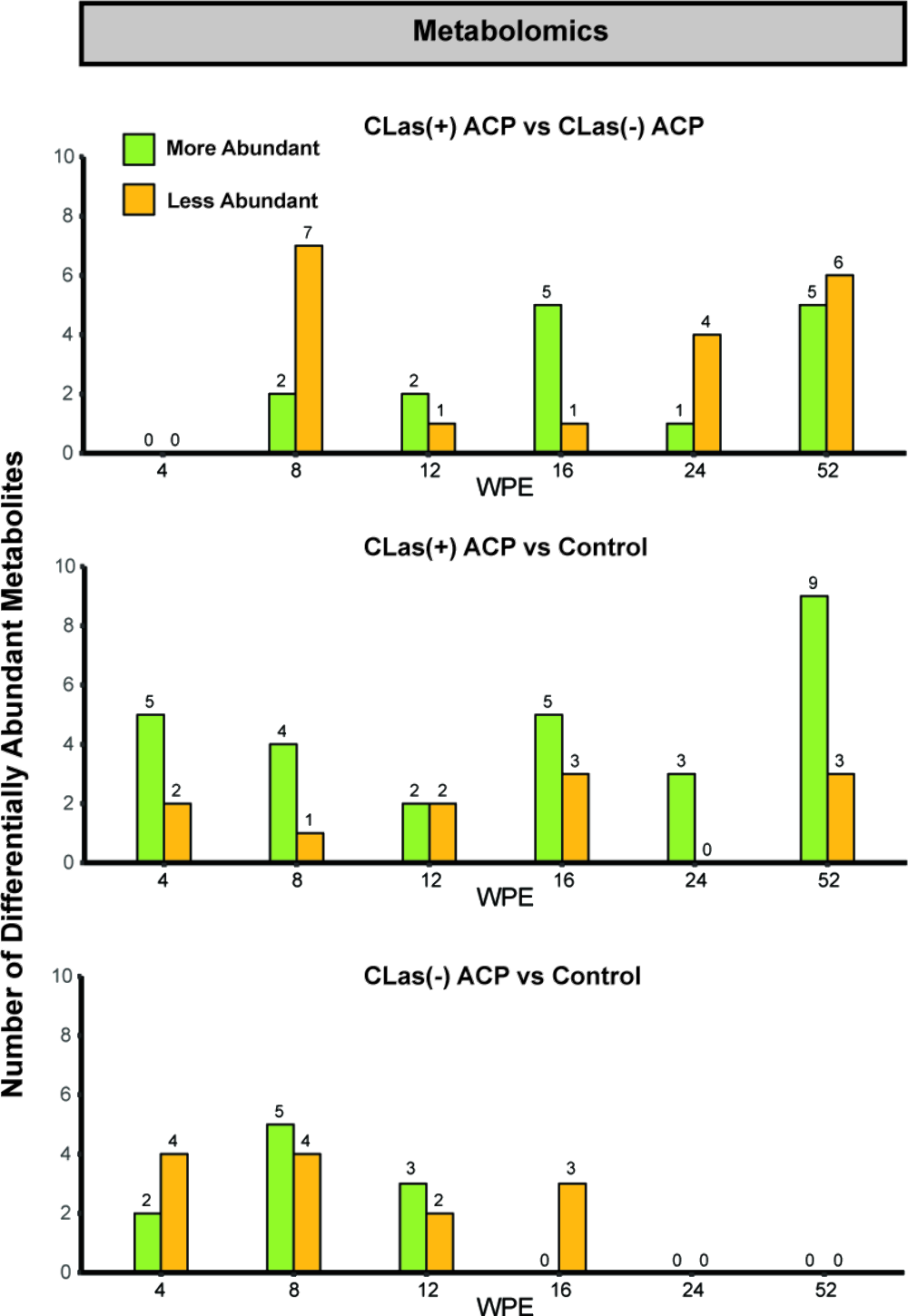
Number of differentially abundant metabolites at 4, 8, 12, and 16 wpe between pairwise comparisons of trees exposed to *C*Las(+) ACP, *C*Las(−) ACP, and nonexposed control trees.

Due to the temporal nature of the metabolite concentrations, Kruskal–Wallis with post hoc Dunn’s multiple comparison tests (FDR ≤ 0.05) was used to identify metabolites with significantly different concentrations between experimental groups at each time point. The total number of differentially abundant metabolites for the time points corresponding to the transcriptomic and proteomic data are summarized in Figure 8. At 4 wpe, trees exposed to *C*Las(+) ACP or *C*Las(−) ACP had no differentially accumulated metabolites, and as with transcriptomic and proteomic data, this suggests that the initial response of the plant is to ACP herbivory rather than to the pathogen. From 4 to 52 wpe, the number of differentially abundant metabolites ranged from zero to 13 (Figure S3), with an average of seven metabolites between trees exposed to *C*Las(+) ACP vs control plants, three metabolites between trees exposed to *C*Las(−) ACP vs control plants, and six metabolites between trees exposed to *C*Las(+) ACP or *C*Las(−) ACP.

Analysis of only the early, asymptomatic-associated time points revealed quinic acid was consistently significantly lower in *C*Las(+) ACP exposed plants relative to control at 4, 8, 12, and 16 wpe, and significantly lower in *C*Las(−) ACP exposed plants relative to control at 8, 12, and 16 wpe, with no difference between trees exposed to *C*Las(+) ACP or *C*Las(−) ACP. Quinic acid is an organic acid intermediate of the shikimate pathway important for the synthesis of aromatic amino acids and other secondary metabolites.^47^ Although an increase in quinic acid in diseased leaves has previously been proposed as a good biomarker for detection of *C*Las during the presymptomatic stage,^48,49^ our results suggest that it is associated with the plant response to ACP herbivory.

Investigation into trends between *C*Las(+) ACP and *C*Las(−) ACP identified the amino acid proline as the only metabolite consistently less abundant in *C*Las(−) ACP exposed leaves at 8, 12, and 16 wpe. No metabolites were consistently differentially abundant between *C*Las(−) ACP exposed leaves and control leaves at 4, 8, 12, and 16 wpe.

Although there may be a few statically significant differences in individual compounds, it is possible to identify shifts in the leaf metabolome using PERMANOVA and subsequent pairwise testing (FDR ≤ 0.05) (Table 2) complemented with visualization by PCA (Figure S4). Multivariate analysis determined that the metabolomes of *C*Las(+) ACP exposed trees are distinguishable from those of control trees at all time points where pairwise testing took place from 4 to 52 wpe. For trees exposed to *C*Las(+) ACP relative to *C*Las(−) ACP, PERMANOVA identified no difference in the leaf metabolome of these groups at 4 wpe; however, the metabolomes of these trees were dissimilar from 8 wpe onward. Interestingly, results of the last pairwise comparison indicated that the metabolomes of *C*Las(−) ACP exposed trees were distinguishable from control trees at 4, 8, 12, and 16 wpe, but not at 20, 24, 28, 32, 36, 40, and 52 wpe. At 44 wpe, plants were sprayed for spider mites prior to sample collection, which may have affected the plant metabolome, and thus we are not considering 44 wpe further. By 20 wpe, few to no psyllids remained on trees as the last psyllid was removed at 21 wpe, meaning these trends coincided with removal of psyllids.

**Table 2:** Metabolomics PERMANOVA and Subsequent Pairwise Testing

	WPE^a^												
Pairwise comparison	4†	8	12†	16	20	24	28†	32	36	40	44	48	52
*C*Las(+) ACP vs control	*	**	**	**	**	**	*	**	*	**	**	–	**
*C*Las(−) ACP vs control	*	**	**	*	ns	ns	ns	ns	ns	ns	**	–	ns
*C*Las(+) ACP vs *C*Las(−) ACP	ns	**	**	**	**	**	*	**	**	**	**	–	**

a *, Significance of FDR ≤ 0.05; **, significance of FDR ≤ 0.01; ns, not significant; †, multivariate homogeneity of variance, an assumption of PERMANOVA, failed, subsequent pairwise testing was not done.

## Discussion

In this study, we explored the tree response to ACP-inoculation with *C*Las using transcriptomic, proteomic, and metabolomic analysis. There was support from all three analyses that the initial plant response during *C*Las infection progression was primarily due to ACP herbivory. At 4 wpe, a substantially lower count of differentially expressed genes and differentially abundant proteins and metabolites was observed between *C*Las(+) ACP and *C*Las(−) ACP exposed trees than between each respective psyllid treatment compared with control trees. However, by 8 weeks, all three analyses revealed the impact of *C*Las on plant metabolism. Interestingly, comparison of the transcriptomes of *C*Las(+) vs *C*Las(−) ACP exposed trees during the four asymptomatic time points identified 17 transcripts that were consistently differentially up- or downregulated. These transcripts are associated with diverse biological functions including the circadian clock (Reveille 1, Adiago protein 3), photosynthesis (α-carbonic anhydrase 1), stress (chaperone protein J, phosphoinositide phospholipase C2 (PLC2), CBL-interacting protein kinase), various cellular processes (transmembrane transporter, lysine-specific demethylase, N-acetyl transferase, NAC containing DNA binding transcription factor), and secondary metabolism (cytochrome P450s).

Plant immunity against pathogens relies on the recognition of conserved microbe-specific structures or molecular motifs known as microbe-associated molecular patterns (MAMPs) or pathogen-associated molecular patterns (PAMPs) that are recognized by plant innate immune systems. Despite previous research that has shown the importance of PAMP-triggered pathways in HLB,^50^ other than the PAMP-associated PLC2 gene, which is believed to play a role in PAMP-triggered immunity as it is rapidly phosphorylated upon exposure to the bacterial flagellin peptide flg22,^51,52^ our study found no evidence of PAMP-associated genes being consistently induced by *C*Las during early infection, suggesting that the expression of these genes may vary in a time-dependent manner.

A growing body of literature is emphasizing the importance of the circadian clock to plant defense.^53^ Reveille 1 (RVE1), a MYB- transcription factor, has been shown to play important roles in both positively regulating auxin production^54^ and chlorophyll biosynthesis^55^ during daylight hours. Starting at 4 wpe, early and consistent downregulation of RVE1 in *C*Las(+) infected leaves may be one of the earliest mechanisms that simultaneously triggers changes in hormone synthesis, photosynthesis, and cellular stress. Interestingly, downregulation of RVE1 has been observed in susceptible varieties relative to tolerant ones, which suggests that expression of RVE1 may play a role in determining susceptibility to *C*Las.^56,57^ However, these results are inconsistent with studies of graft-inoculated *C*Las, where RVE1 is downregulated in infected and upregulated in susceptible varieties relative to mock inoculated controls.^58^

Three transcripts for cytochrome P450 monooxygenases were upregulated in *C*Las(+) ACP exposed trees relative to *C*Las(−) ACP exposed trees that included one transcript corresponding to cytochrome P450 71A1 and two transcripts corresponding to cytochrome P450 83B1, known to be associated with biosynthesis of glucosinolates and callose deposition upon pathogen attack.^59^ Cytochrome P450s are involved in biosynthesis of secondary metabolites like flavonoids, and in detoxification.^60^ Altered expression of cytochrome P450 genes has previously been reported in *C*Las-infected trees^56^ along with increased callose deposition that ultimately inhibits transport of nutrients in the phloem.^61,62^ Interestingly, it was recently shown that the genome of *C*Las encodes a putative virulence factor capable of interacting with a cytochrome P450 71A1-like protein,^63^ suggesting that citrus cytochrome P450s are important targets of the pathogen during colonization.

Proteome comparisons of *C*Las(+) ACP versus *C*Las(−) ACP exposed trees identified only one protein, starch synthase, as consistently differentially accumulated at the asymptomatic time points. Starch synthase was subtly less abundant in *C*Las(+) ACP exposed trees at 4 wpe, but became more abundant in these plants starting at 8 wpe and continued to be higher with increasing fold changes at each subsequent time point until the end of the study at 52 wpe (Table S3). Accumulation of starch via an increase in starch synthase is a characteristic symptom of HLB and contributes to blocking transport of nutrients throughout the plant resulting in localized nutrient starvation.^62,64^ Starch content in herbivore-attacked leaves tends to decrease as plants catabolize these energy-rich compounds to offset the cost of plant defense.^65^ The lack of a coordinated defense in the plant is emphasized by the consistently downregulated α-carbonic anhydrase in *C*Las-infected trees. Carbonic anhydrase 1 is associated with suppression of salicylic acid-dependent defense and has been shown to be downregulated in response to pathogen infection.^66^ Interestingly, salicylic acid-related metabolites were reported to be reduced in tolerant citrus varieties.^67^

Although no metabolites were found to be differentially accumulated at 4 wpe between leaves of *C*Las(+) ACP or *C*Las(−) ACP exposed trees, proline was found to be significantly higher in the *C*Las(+) ACP exposed leaves at 8, 12, and 16 wpe. Proline is a stress-induced metabolite shown to accumulate in response to pathogen infection,^68^ herbivory,^69^ and known to play important roles in stabilizing cell membranes, free radical detoxification, and aiding osmotic balance.^70^ Since proline concentration is regulated by a variety of biotic and abiotic factors, proline alone has been suggested as a poor HLB-specific biomarker.^71^ Nonetheless, trends have emerged with respect to proline levels in HLB-diseased leaves. Proline concentration has previously been associated with elevated levels in symptomatic leaves relative to *C*Las-negative leaves,^8,71^ but no significant difference was observed between symptomatic and asymptomatic leaves.^72^ Additionally, trees graft-inoculated with *C*Las have elevated proline levels relative to those exposed to *C*Las(−) ACP herbivory.^8^ Our results add to this literature by showing that leaves of trees inoculated with *C*Las by ACP exhibit higher proline concentrations in early, potentially asymptomatic infection relative to *C*Las(−) ACP exposed leaves and may provide an early marker of infection.

Previous research has suggested a density-dependent effect of ACP feeding on citrus metabolism.^7^ We report the first evidence that citrus returns to a normal metabolic signature shortly after ACP herbivory ceases. Starting 2 weeks postintroduction, the process of removing ACP began and continued until the last psyllid in this study was extracted at ∼21 wpe. Given that it took ∼21 weeks to remove all of the psyllids, we may have had a second generation of psyllids. However, their impact was likely minimal, as at 16 wpe, no DEGs between leaves of trees exposed to *C*Las(−) ACP and control trees were observed, and by 24 weeks no differences in the metabolome were observed between leaves of trees exposed to *C*Las(−) ACP and control trees. Indeed, except for 44 wpe when trees were sprayed for spider mites prior to sample collection, the metabolomes of *C*Las(−) ACP and control trees were indistinguishable starting at 20 wpe (PERMANOVA, Table 2). Additionally, except for 24 wpe when trees were moved from insect rearing chambers into the greenhouse, the number of differentially abundant proteins between the *C*Las(−) ACP and nonexposed trees decreased with time, thus movement of the trees from the growth chambers to the greenhouse had minimal impact on our results. While plant growth may be limited under ACP-induced stress, it appears this phenomenon is temporary, and citrus is able to return to a normal metabolic state after psyllids are removed. This has important implications as it suggests that citrus plants are not permanently metabolically altered by short-term psyllid feeding and further suggests that the impact of the pathogen can be differentiated from the impact of insect feeding.

The information in this study sheds light on the variation of plant response to both ACP and *C*Las over time. Additionally, we observed that trees exposed to a phloem-feeding insect return to a normal metabolic state shortly after herbivory ceases. In addition to clarifying the impact of ACP on citrus metabolism, this observation also improves our understanding of ecological relationships between phloem-feeding insects and their host plants. The results of this study provide a broader understanding of plant–microbe and plant–vector interactions.

## Data Availability

RNaseq data can be accessed in the SRA Database under entry PRJNA985365. Proteomics data can be accessed from Dryad at 10.25338/B83H1Z. Metabolomics data can be accessed from Dryad at 10.25338/B83P9Q.

## Supporting Information Available

Supplementary Methods: Preparation of leaf tissue for transcriptomics and metabolomics; RNA extraction, library preparation, and sequencing; citrus leaf protein extraction and precipitation; peptide sample preparation; gel analysis; reduction/alkylation/trypsin digestion; C18 column cleanup; mass spectrometry data acquisition; sample preparation and proton NMR data acquisition for metabolomics. Supplementary Tables: Table S1. Average Ct values for qPCR detection of *C*Las in citrus leaves. Trees were designated as “positive” for *C*Las or “negative” for *C*Las using the APHIS-PPQ Ct cutoff of Ct ≤ 36. Trees exposed to *C*Las(+) ACP underwent analysis at 4, 6, 8, 12, 16, 20, 50, and 52 wpe*. Trees not exposed (“control”) or exposed to *C*Las(−) ACP underwent analysis at 4, 6, 50, and 52 wpe. Dashes indicate times when data was not obtained. Bold plant IDs with corresponding experimental group information indicate the subset of plants that underwent transcriptomic and proteomic analyses. Table S2. Average total reads for each treatment group obtained after cleaning and average percent of reads mapped to the *Citrus sinensis* genome. Table S3. Differential abundance of statistically significant starch synthase proteins between trees exposed to *C*Las(+) ACP or *C*Las(−) ACP as determined by Fisher’s exact tests. FDR: False discovery rate corrected *p*-value. Supplementary Figures: Figure S1. Proteomics principal component analysis (PCA) and corresponding loadings plot showing the top 20 variables contributing to principal component 1 (PC1) and principal component 2 (PC2) for 0, 4, 8, 12, 16, 24, and 52 wpe (A-G). Figure S2. Log10 transformed aspartate concentrations for all experimental groups from 0 (baseline) to 52 wpe. Median and interquartile ranges are shown. Figure S3. Venn diagrams showing the total number of differentially accumulated metabolites between pairwise comparisons and the number of overlapping metabolites. Figure S4. Metabolomics principal component analysis (PCA) and corresponding loadings plot showing the top 20 variables contributing to principal component 1 (PC1) and principal component 2 (PC2) for 0, 4, 8, 12, 16, 24, and 52 wpe (A-G). (PDF)

## Abbreviations

ACP: Asian citrus psyllid (*Diaphorina citri* Kuwayama)
*C*Las: *Candidatus* Liberibacter asiaticus
DAPs: Differentially abundant proteins
DEGs: Differentially expressed genes
FC: Fold change
FDR: False discovery rate
HLB: Huanglongbing
MAMPs: Microbe-associated molecular patterns
PAMPs: Pathogen-associated molecular patterns
PLC2: Phosphoinositide phospholipase C2
qPCR: Quantitative polymerase chain reaction
RVE1: Reveille 1
wpe: Weeks postexposure
