# Supplementary material for: Longitudinal Transcriptomic, Proteomic, and Metabolomic Response of Citrus sinensis to Diaphorina citri Inoculation of Candidatus Liberibacter asiaticus
Source: J Proteome Res. 2024 Feb 19;23(8):2857–69. doi: 10.1021/acs.jproteome.3c00485 (PMC11301674; doi:10.1021/acs.jproteome.3c00485)
Supplement: Supplementary file 1 — pr3c00485_si_001.pdf [file pr3c00485_si_001.pdf]

Supplementary Information

Longitudinal Transcriptomic, Proteomic, and Metabolomic Response of *Citrus sinensis* to *Diaphorina citri* Inoculation of *Candidatus* Liberibacter asiaticus

Rachel L. Lombardi, John S. Ramsey, Jaclyn E. Mahoney, Michael J. MacCoss, Michelle L. Heck and Carolyn M. Slupsky

Contents:

Supplementary methods:

Preparation of Leaf Tissue for Transcriptomics and Metabolomics

RNA Extraction, Library Preparation, and Sequencing

Citrus Leaf Protein Extraction and Precipitation

Peptide Sample Preparation

Gel Analysis

Reduction/alkylation/trypsin Digestion

C18 Column Cleanup

Mass Spectrometry Data Acquisition

Sample Preparation and Proton NMR Data Acquisition for Metabolomics

Supplementary Tables:

Table S1. Average Ct values for qPCR detection of *C*Las in citrus leaves. Trees were designated as "positive" for *C*Las or "negative" for *C*Las using the APHIS-PPQ Ct cutoff of Ct ≤ 36. Trees exposed to *C*Las(+) ACP underwent analysis at 4, 6, 8, 12, 16, 20, 50 and 52 wpe*. Trees not exposed ("control") or exposed to *C*Las(-) ACP underwent analysis at 4, 6, 50, and 52 wpe. Dashes indicate times when data was not obtained. Bold plant IDs with corresponding experimental group information indicate the subset of plants that underwent transcriptomic and proteomic analyses.

Table S2. Average total reads for each treatment group obtained after cleaning and average percent of reads mapped to the *Citrus sinensis* genome.

Table S3. Differential abundance of statistically significant starch synthase proteins between trees exposed to *C*Las(+) ACP or *C*Las(-) ACP as determined by Fisher's exact tests. FDR: False discovery rate corrected p-value.

Supplementary Figures:

Figure S1. Proteomics principal component analysis (PCA) and corresponding loadings plot showing the top 20 variables contributing to principal component 1 (PC1) and principal component 2 (PC2) for 0, 4, 8, 12, 16, 24, and 52 wpe (A-G).

Figure S2. Log10 transformed aspartate concentrations for all experimental groups from 0 (baseline) to 52 wpe. Median and interquartile ranges are shown.

Figure S3. Venn diagrams showing the total number of differentially accumulated metabolites between pairwise comparisons and the number of overlapping metabolites.

Figure S4. Metabolomics principal component analysis (PCA) and corresponding loadings plot showing the top 20 variables contributing to principal component 1 (PC1) and principal component 2 (PC2) for 0, 4, 8, 12, 16, 24, and 52 wpe (A-G).

SUPPLEMENTARY METHODS

Preparation of Leaf Tissue for Transcriptomics and Metabolomics

In preparation for cryogenic grinding of leaves, all disposable supplies were purchased RNase-free and were pre-chilled prior to the start of grinding. RNaseZap (Thermo Fisher Scientific, Waltham, MA) was used to decontaminate the workspace and gloves. Foil packets containing leaves were unwrapped on dry ice and a dry KimWipe (Fisher Scientific) was used to quickly remove any remaining greenhouse debris. Leaves were cryoground using a pre-chilled mortar and pestle with liquid nitrogen. Using a sterile spatula, a maximum of 100 mg of frozen ground tissue was transferred into a 1.5 mL microcentrifuge tube for RNA extraction for transcriptomic analysis. Additional tissue was used to fill a 2 mL microcentrifuge tube for metabolomic analysis. All tubes were stored at -80 °C prior to further sample preparation.

RNA Extraction, Library Preparation, and Sequencing

RNA extraction was carried out using Qiagen RNeasy Plant Mini Kit (cat#: 74904) using Buffer RLT with the addition of a DNase digestion step (RNase-Free DNase Set, Qiagen, cat#: 79254) as described in the user manual.

Total RNA purity of the eluent was determined using a NanoDrop ND-1000 spectrophotometer (Thermo Scientific), quality was assessed using a Bioanalyzer 2100 with the RNA 6000 Nano Kit (RIN $\geq$ 7; Agilent Technologies, cat#: 5067-1511), and quantification was carried out using a Qubit Fluorometer 3.0 (Life Technologies, Carlsbad, CA) with the Qubit RNA HS Assay Kit (Thermo-Fisher, cat#: Q32852). RNA concentrations between samples were normalized using RNase/DNase free water prior to submission to the UC Davis Sequencing Core for library preparation.

Strand-specific and barcode indexed RNA-seq libraries were generated from 700 ng following poly-A enrichment using the KAPA mRNA-seq Hyper kit (KAPA Biosystems, Cape Town, South Africa) following the manufacturer's instructions. Micro-capillary gel electrophoresis on a Bioanalyzer 2100 was used to verify the fragment size distribution of the cDNA libraries. The libraries were quantified using a Qubit fluorometer and pooled in equimolar ratios. qPCR was carried out using the KAPA Library Quant kit (KAPA Biosystems) to quantify the pooled libraries prior to sequencing on four lanes of an Illumina HiSeq 4000 (Illumina, San Diego, CA) with paired-end 150 bp reads.

Citrus Leaf Protein Extraction and Precipitation

10x volume (10 mL/1 g) of precipitation solvent (10% trichloroacetic acid in acetone with 2% β-mercaptoethanol) was added to 0.5-1 g of ground leaf samples. Samples were vortexed, homogenized for 30 s (Kinematica Polytron), sonicated for 30 s at 15% amplitude (Branson Digital Sonifier), and stored overnight at −20 °C to allow proteins to precipitate. The resulting protein pellet was washed three times with 10 mL of ice-cold acetone. For the third acetone wash, the acetone volume added was 10x (10 mL/1 g) of the original weighed tissue, and the protein slurry was divided into 1.5 mL aliquots, which were pelleted, dried separately, and stored at −80 °C.

Peptide Sample Preparation

Protein pellets were resuspended in 500 μL of protein reconstitution solvent (8 M urea, 50 mM TEAB, in water), vortexed, and incubated overnight at room temperature with shaking at 1400 rpm and flea pellet added (Tomy microtube mixer MT-360). Samples were centrifuged at 16,100 × g for 10 min, and the supernatant was collected. Sample protein concentration was measured using the Quick Start Bradford protein assay (Bio-Rad, Hercules, CA).

Gel Analysis

Gel electrophoresis was used as a quality control check to validate the Bradford results. Ten µg of protein sample was adjusted to a volume of 25 µL using phosphate buffered saline solution. 25 µL of 2x Laemmli Sample Buffer (Bio-Rad) containing 5% β-mercaptoethanol was added to each protein sample. Samples were incubated at 70 °C for 10 minutes and run on a 10% Mini-PROTEAN TGX Pre-cast gel (Bio-Rad) at 80V for 2 hours, using SDS-PAGE running buffer and a Precision Plus Protein Kaleidoscope standard (Bio-Rad). After running, the gels were removed from their casing and transferred to a plastic container to stain using Invitrogen NOVEX Colloidal Blue Staining Kit, following instructions for Tris-Glycine gels (Life Technologies). Gels were stained overnight on a rocker. To de-stain, the gels were rocked in Milli-Q water for a few hours, replacing water as needed. Once de-stained, gels were scanned, and samples were compared.

Reduction/alkylation/trypsin Digestion

Protein samples were reduced with tris carboxyethyl phosphine (TCEP, 10 mM): 5 µL of 200 mM TCEP in water was added to 40 µg of protein in 95 µL of 100 mM TEAB and incubated at 30 °C for 1 h. Cysteine alkylation was performed by adding 5 µL of 375 mM iodoacetamide to each sample. Samples were vortexed, briefly centrifuged at room temperature to pull down condensation, and incubated for 1 h at room temperature in the dark. If necessary, 100 mM TEAB was added to samples prior to trypsin digestion to reduce urea concentration to <1 M. Care was taken to ensure the urea concentration and trypsin digestion reaction volumes were the same across all samples.

Sequencing grade modified trypsin (Promega, Madison, WI) was added to each sample (trypsin/protein ratio of 1:40 by weight), and samples were vortexed, briefly centrifuged at room temperature to pull down condensation, and incubated overnight at 30 °C.

C18 Column Cleanup

Dried trypsin-digested samples were resuspended in 380 µL of 0.1% formic acid in water. Samples were acidified to a pH ≤ 3 by adding 5 µL of full-strength formic acid, and pH was tested via pH paper. Waters Sep-Pak C18 1 cc vacuum cartridges (cat#: WAT054955) were used with a Phenomenex vacuum manifold, with pressure kept between 4 and 5 inHg. Columns were conditioned with 3 mL of 100% acetonitrile, followed by 3 mL of 0.1% formic acid. Columns were briefly dried to remove all liquid from the column, and then samples were added and run through. Columns were then washed with 3 mL of 0.1% formic acid and briefly dried to remove all liquid from the column. Samples were eluted off the column into a collection tube by adding 500 µL of 80:20 (v/v) acetonitrile/0.1% formic acid. The column was dried to ensure that all eluent was collected. Cleaned samples were speed vacuumed (Labconco Centrivap concentrator equipped with a Savant VP100 pump) at room temperature for 1–3 h and stored at −80 °C before liquid chromatography–mass spectrometry (LC–MS) analysis.

Mass Spectrometry Data Acquisition

All mass spectrometry was performed on a Fusion Exploris (Thermo Fisher Scientific) mass spectrometer with a Thermo Easy-nLC HPLC with autosampler. The dried tryptic peptides (~40 µg) were solubilized in 60 µL of loading buffer. The loading buffer was comprised of 0.1% trifluoroacetic acid, 2% acetonitrile in water, and 15 fmol/µL of a peptide standard (Pierce PRTC). Sample volumes of 3 µL were injected via the autosampler onto a 150-µm Kasil fritted

trap (Dr. Maisch Reprosil-Pur 120 C18-AQ 3 µm beads, 2 cm x 150 µm) at a flow rate of 2 µL/min. After loading and desalting using a total volume of 8 µL of loading buffer, the trap was brought on-line with a fritted column (100-µm inner diameter) and packed to a length of 30 cm with the same Dr. Maisch beads. The outlet of the column was attached to an empty pulled tip (20 µm ID pulled to 10 µm) via a zero dead volume connector. The column and trap were mounted to a nanospray ion source (CorSolutions, Ithaca, NY) heated to 50 °C, and placed in line with the HPLC pump. Peptides were eluted off the column using a gradient of 0-36% acetonitrile in 0.1% formic acid over 90 minutes, followed by 36-60% acetonitrile over 10 minutes at a flow rate of 450 nL/min.

The mass spectrometer was operated using electrospray ionization (2 kV) with the heated transfer tube at 300 °C. For data dependent acquisition (DDA), one orbitrap mass spectrum (m/z 395-1005) was acquired with multiple orbitrap MS/MS spectra every three seconds or less. The resolution for MS in the orbitrap was 60,000 at m/z 200, and 15,000 for MS/MS. The automatic gain control targets for MS and MS/MS were 300% and 100%. The maximum fill times were set automatically, depending on the resolution settings. The quadrupole isolation width was 1.4 m/z and HCD collision energy was 27%. The precursor ion threshold intensity was set to 5e3 to trigger an MS/MS acquisition. Furthermore, MS/MS acquisitions were allowed for precursor charge states of 2-4. Dynamic exclusion (including all isotope peaks) was set for 20 seconds using monoisotopic precursor selection with a mass error of 10 ppm.

Sample Preparation and Proton NMR Data Acquisition for Metabolomics

Following lyophilization (Labconco FreeZone Plus) of the frozen ground leaf material for 72 hr, 65 mg of dried leaf material was transferred to 2 mL tubes. One 3.5 mm glass bead was added to

each tube before beating (Biospec Mini-BeadBeater 16) for 2 minutes to ensure even particle size. 10 mM phosphate buffer (pH 6.8) pre-heated to 90 °C was added to the ground tissue in a 1:20 (w/v) ratio based on each sample's dry weight. Tubes were immediately mixed for 15 min at 90 °C at 1000 rpm (Eppendorf ThermoMixer C) and then centrifuged at 4 °C for 15 min at 14,000 RCF. A total of 750 µL of supernatant was collected, transferred to a clean 1.5 mL tube, and centrifuged at 4 °C for 15 min at 14,000 RCF. To 585 µL of supernatant, 65 µL of 4.97 mM 4,4-dimethyl-4-silapentane-1-sulfonic acid-d6 (DSS-d6) was added as an internal standard and vortexed. Of this mixture, 600 µL was transferred to 5 mm NMR tubes and stored at 4 °C until NMR data acquisition which took place within 24 hours of NMR sample preparation.

^{1}H NMR data acquisition was performed using a Bruker Advance 600 MHz NMR spectrometer equipped with a SampleJet using the Bruker "noesypr1d" (RD-90°-t-90°-tm-ACQ) acquisition parameters as previously described[1, 2]. Identification and quantification of 27 metabolites was accomplished using Chenomx NMR suite v8.31 (Chenomx Inc., Edmonton, Alberta, Canada), and the resulting concentrations were corrected for dilution based on dry leaf weight.

Table S1. Average Ct values for qPCR detection of *C*Las in citrus leaves. Trees were designated as “positive” for *C*Las or “negative” for *C*Las using the APHIS-PPQ Ct cutoff of Ct ≤ 36. Trees exposed to *C*Las(+) ACP underwent analysis at 4, 6, 8, 12, 16, 20, 50 and 52 wpe*. Trees not exposed (“control”) or exposed to *C*Las(-) ACP underwent analysis at 4, 6, 50, and 52 wpe. Dashes indicate times when data was not obtained. Bold plant IDs with corresponding experimental group information indicate the subset of plants that underwent transcriptomic and proteomic analyses.

Group	Plant ID	4 wpe	6 wpe	8 wpe	//	12 wpe	//	16 wpe	//	20 wpe	//	50 wpe	52 wpe
CLas(+) ACP Exposed Trees	**3**	**37.39**	**35.11**	**28.43**		**27.19**		**28.25**		**22.17**		**24.61**	**20.74**
	4	36.89	37.20	35.68		34.43		17.75		36.89		23.24	19.85
	**10**	**28.04**	**34.84**	**35.70**		**27.50**		**31.79**		**29.84**		**22.61**	**21.18**
	15	33.09	35.75	-		34.78		23.65		20.08		24.45	20.44
	**20**	**35.22**	**37.61**	**35.71**		**21.90**		**29.16**		**20.19**		**23.97**	**20.55**
	24	34.48	34.24	40.00		32.01		18.40		21.82		22.89	20.92
	25	34.13	37.10	40.00		19.53		34.69		31.94		22.51	22.49
	28	36.11	37.12	34.38		36.39		34.56		31.20		21.30	20.46
	30	35.34	34.87	40.00		36.49		35.59		21.09		21.15	19.40
	31	32.80	33.72	34.14		19.48		19.69		20.80		24.32	23.36
	**33**	**25.29**	**23.36**	**40.00**		**20.68**		**19.49**		**22.17**		**21.90**	**21.23**
	**35**	**36.99**	**23.27**	**30.62**		**20.44**		**19.28**		**24.13**		**21.11**	**21.19**
CLas(-) ACP Exposed Trees	**8**	**40.00**	**40.00**	**-**		**-**		**-**		**-**		**40.00**	**40.00**
	9	36.75	40.00	-		-		-		-		40.00	36.01
	13	36.73	40.00	-		-		-		-		40.00	40.00
	14	40.00	37.28	-		-		-		-		38.94	40.00
	16	36.95	40.00	-		-		-		-		40.00	40.00
	**17**	**40.00**	**40.00**	**-**		**-**		**-**		**-**		**40.00**	**40.00**
	19	38.16	40.00	-		-		-		-		40.00	40.00
	**23**	**40.00**	**40.00**	**-**		**-**		**-**		**-**		**40.00**	**40.00**
	26	37.13	37.73	-		-		-		-		36.94	40.00
	27	40.00	37.19	-		-		-		-		40.00	34.92
	**32**	**36.99**	**40.00**	**-**		**-**		**-**		**-**		**40.00**	**40.00**
	**36**	**35.56**	**40.00**	**-**		**-**		**-**		**-**		**37.42**	**40.00**
Control Trees	1	36.28	40.00	-		-		-		-		36.85	40.00
	**2**	**40.00**	**40.00**	**-**		**-**		**-**		**-**		**40.00**	**40.00**
	5	40.00	36.48	-		-		-		-		40.00	32.51
	6	37.75	40.00	-		-		-		-		40.00	40.00
	7	40.00	40.00	-		-		-		-		40.00	34.49
	**11**	**40.00**	**37.19**	**-**		**-**		**-**		**-**		**40.00**	**40.00**
	12	40.00	40.00	-		-		-		-		40.00	40.00
	18	40.00	37.66	-		-		-		-		37.41	34.83
	21	40.00	40.00	-		-		-		-		40.00	40.00
	22	40.00	37.13	-		-		-		-		37.62	36.87
	**29**	**40.00**	**38.07**	**-**		**-**		**-**		**-**		**40.00**	**40.00**
	**34**	**40.00**	**36.98**	**-**		**-**		**-**		**-**		**40.00**	**40.00**

*wpe: weeks post-exposure

Table S2. Average total reads for each treatment group obtained after cleaning and average percent of reads mapped to the *Citrus sinensis* genome.

Experimental Group	Average Total Reads	Average Percent of Mapped Reads
Control	15,252,175	90%
CLas(-) ACP	15,281,599	89%
CLas(+) ACP	15,419,060	87%

Table S3. Differential abundance of statistically significant starch synthase proteins between trees exposed to *C*Las(+) ACP or *C*Las(-) ACP as determined by Fisher's exact tests. FDR: False discovery rate corrected p-value.

WPE	Accession Number	FDR	Fold Change	Average Fold Change
4	orange1.1t00566.2	< 0.00010	0.5	-
8	orange1.1t00566.2	< 0.00010	2.2	-
12	orange1.1t00566.2	< 0.00010	2.5	2.4
	orange1.1t00566.7	< 0.00010	2.5	
	orange1.1t00566.9	< 0.00010	2.2	
16	orange1.1t00566.2	< 0.00010	3.5	
24	orange1.1t00566.2	< 0.00010	4.4	4.4
	orange1.1t00566.7	< 0.00010	4.5	
	orange1.1t00566.9	< 0.00010	4.2	
52	orange1.1t00566.2	< 0.00010	5	5
	orange1.1t00566.5	< 0.00010	5	

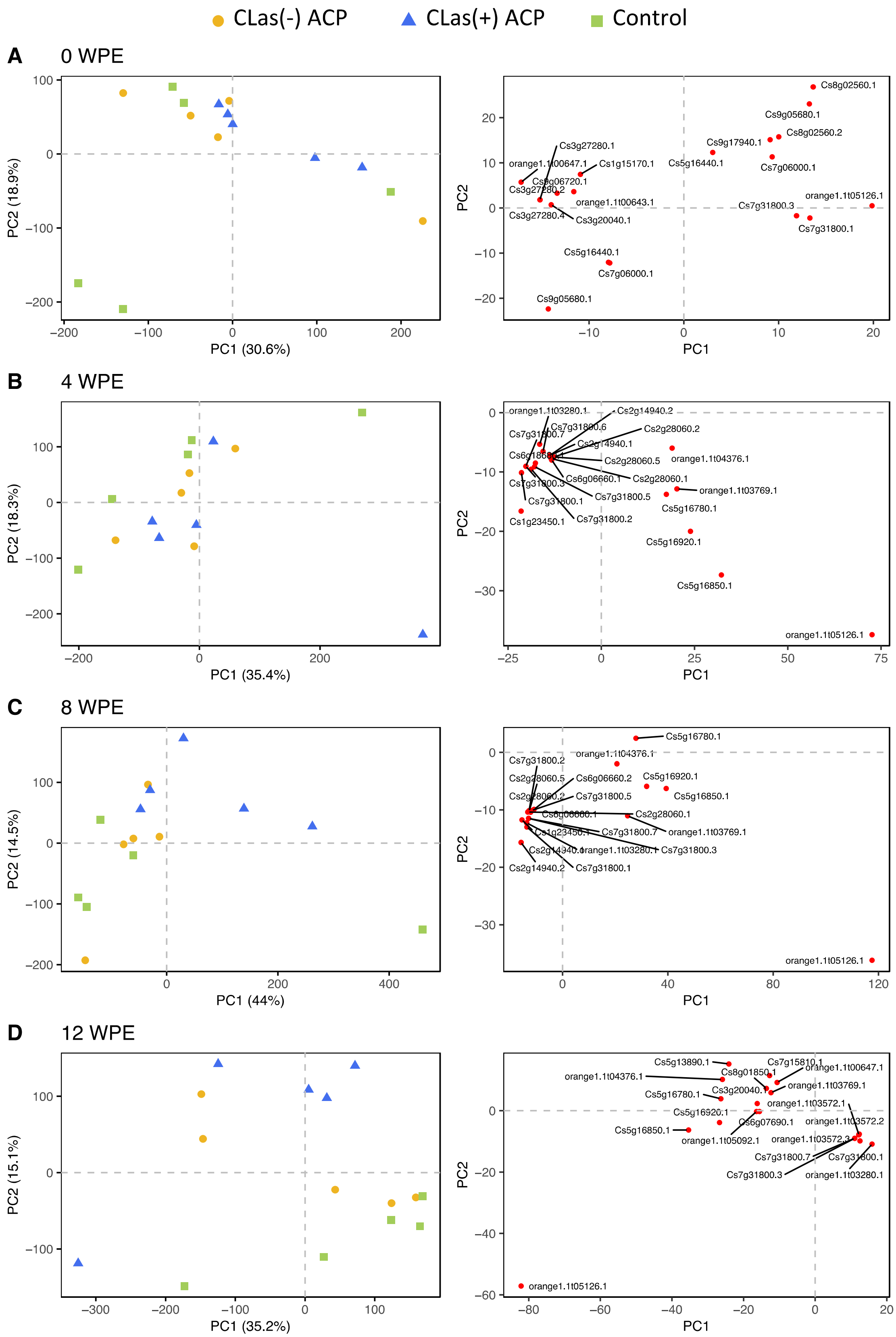

CLas(-) ACP
CLas(+) ACP
Control
A
0 WPE
PC1 (30.6%)
PC2 (18.9%)
PC1
PC2
Cs8g02560.1
Cs9g05680.1
Cs8g02560.2
Cs9g17940.1
Cs5g16440.1
Cs7g06000.1
Cs3g27280.1
orange1.1t00647.1
Cs1g15170.1
Cs9g06720.1
Cs3g27280.2
orange1.1t00643.1
Cs3g27280.4
Cs3g20040.1
Cs7g31800.3
orange1.1t05126.1
Cs7g31800.1
Cs5g16440.1
Cs7g06000.1
Cs9g05680.1
B
4 WPE
PC1 (35.4%)
PC2 (18.3%)
orange1.1t03280.1
Cs2g14940.2
Cs7g31800.6
Cs7g31800.7
Cs2g28060.2
Cs2g14940.1
Cs6g18600.1
Cs2g28060.5
orange1.1t04376.1
Cs7g31800.3
Cs6g06660.1
Cs2g28060.1
Cs7g31800.1
Cs7g31800.5
orange1.1t03769.1
Cs5g16780.1
Cs1g23450.1
Cs7g31800.2
Cs5g16920.1
Cs5g16850.1
orange1.1t05126.1
C
8 WPE
PC1 (44%)
PC2 (14.5%)
Cs5g16780.1
orange1.1t04376.1
Cs7g31800.2
Cs2g28060.5
Cs6g06660.2
Cs5g16920.1
Cs2g28060.2
Cs7g31800.5
Cs5g16850.1
Cs6g06660.1
Cs2g28060.1
Cs1g23450.1
Cs7g31800.7
orange1.1t03769.1
Cs2g14940.1
orange1.1t03280.1
Cs7g31800.3
Cs2g14940.2
Cs7g31800.1
orange1.1t05126.1
D
12 WPE
PC1 (35.2%)
PC2 (15.1%)
Cs5g13890.1
Cs7g15810.1
orange1.1t04376.1
Cs8g01850.1
orange1.1t00647.1
Cs5g16780.1
Cs3g20040.1
orange1.1t03769.1
orange1.1t03572.1
Cs5g16920.1
Cs6g07690.1
orange1.1t03572.2
Cs5g16850.1
orange1.1t05092.1
orange1.1t03572.3
Cs7g31800.7
Cs7g31800.1
Cs7g31800.3
orange1.1t03280.1
orange1.1t05126.1

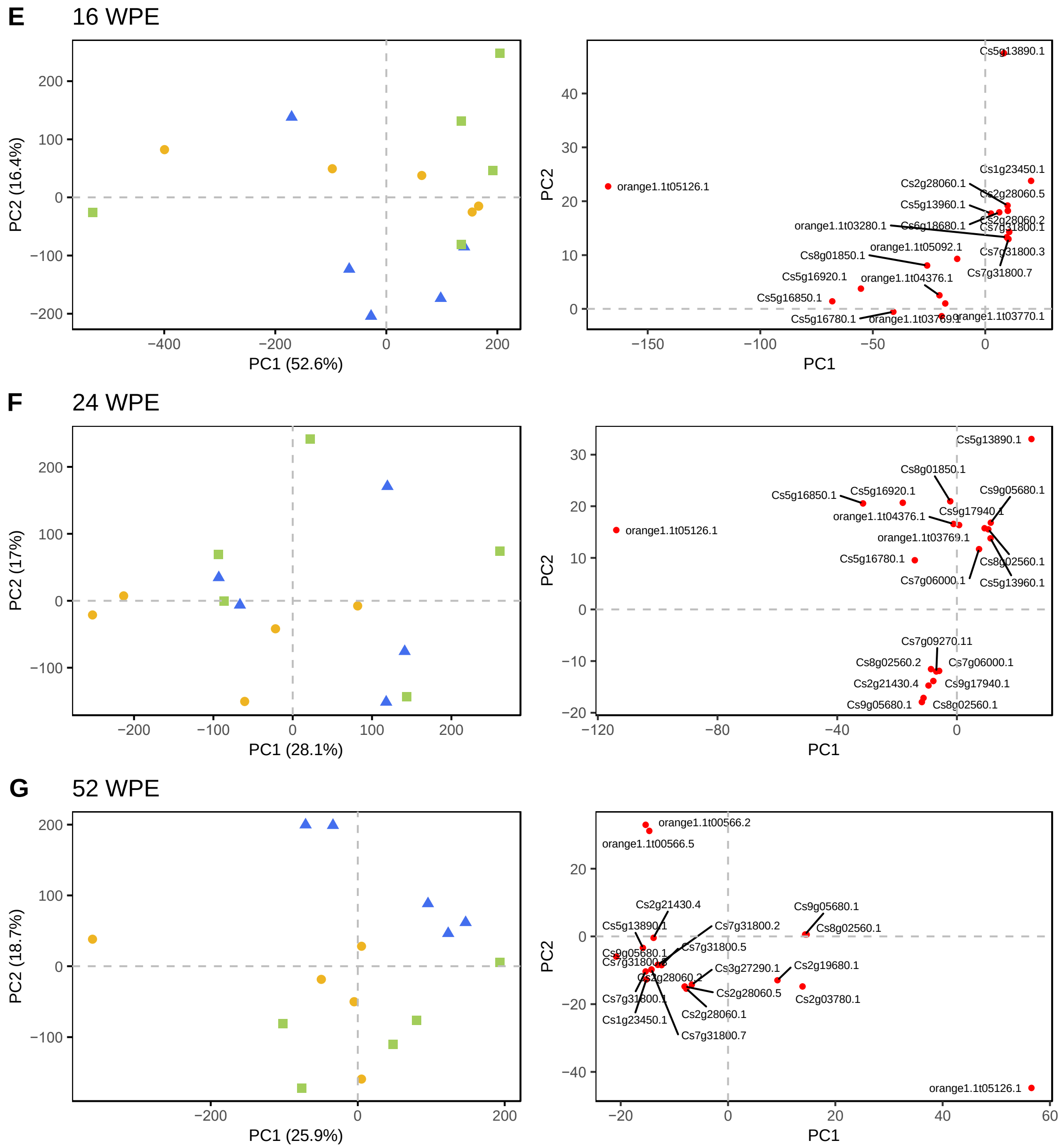

Figure S1. Proteomics principal component analysis (PCA) and corresponding loadings plot showing the top 20 variables contributing to principal component 1 (PC1) and principal component 2 (PC2) for 0, 4, 8, 12, 16, 24, and 52 wpe (A-G).

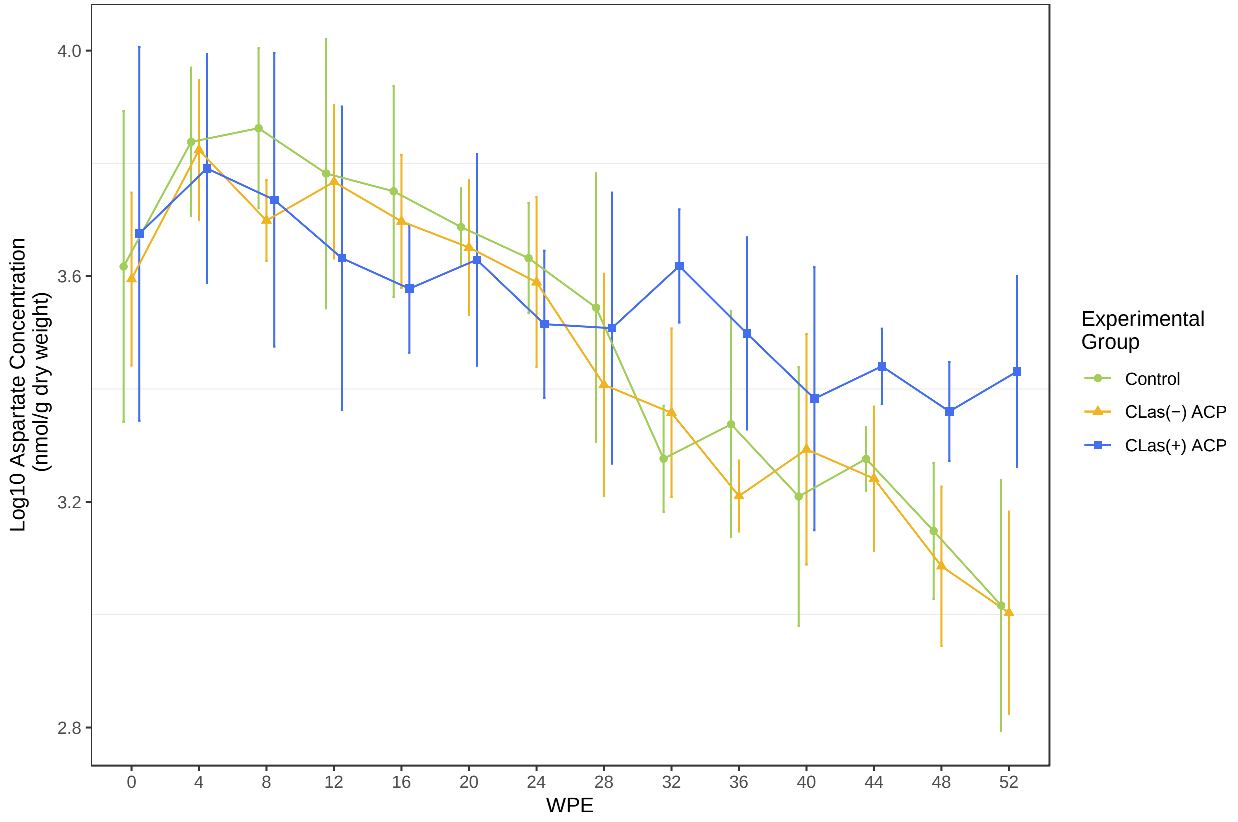

Figure S2. Log10 transformed aspartate concentrations for all experimental groups from 0 (baseline) to 52 wpe. Median and interquartile ranges are shown.

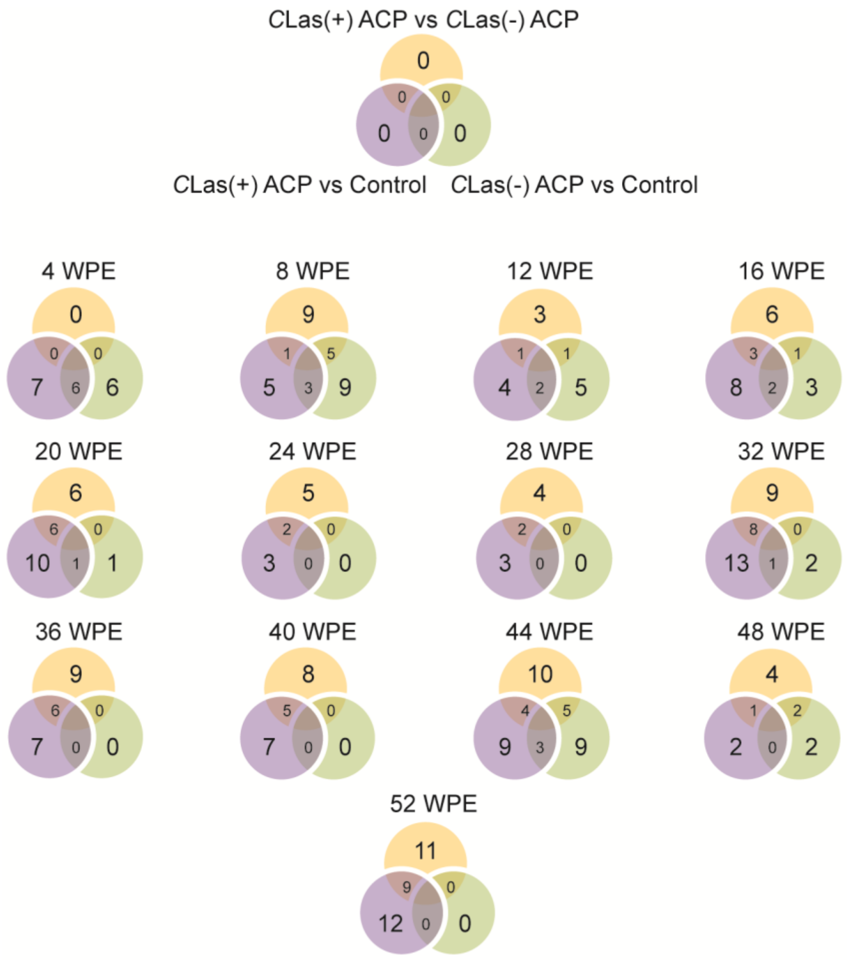

Figure S3. Venn diagrams showing the total number of differentially accumulated metabolites between pairwise comparisons and the number of overlapping metabolites.

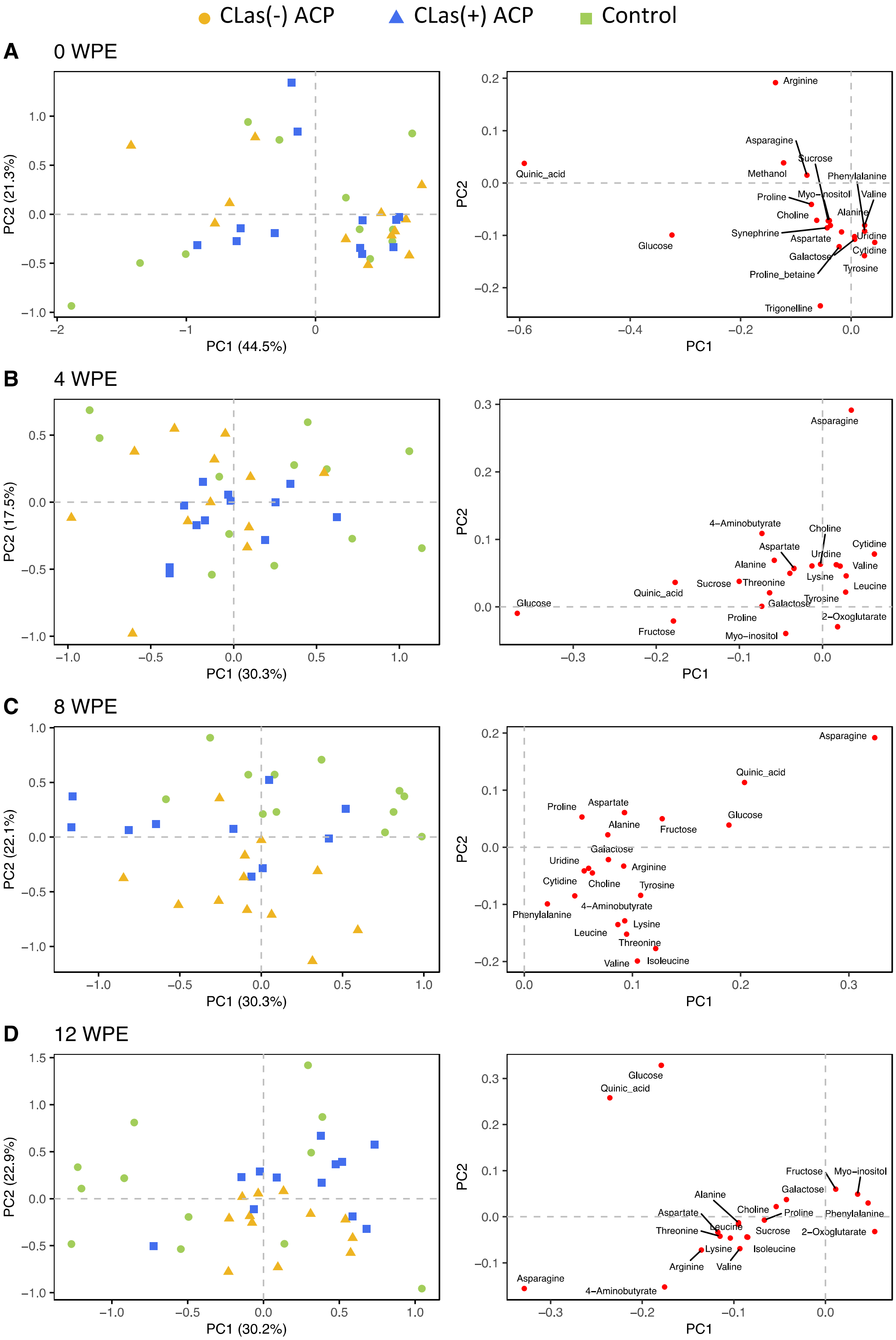
CLas(-) ACP
CLas(+) ACP
Control
A
0 WPE
PC2 (21.3%)
PC1 (44.5%)
Arginine
Asparagine
Sucrose
Methanol
Phenylalanine
Quinic_acid
Proline
Myo−inositol
Valine
Choline
Alanine
Synephrine
Aspartate
Uridine
Glucose
Galactose
Cytidine
Tyrosine
Proline_betaine
Trigonelline
PC2
PC1
B
4 WPE
PC2 (17.5%)
PC1 (30.3%)
Asparagine
4−Aminobutyrate
Choline
Aspartate
Cytidine
Uridine
Alanine
Valine
Lysine
Quinic_acid
Sucrose
Threonine
Leucine
Tyrosine
Glucose
Galactose
2−Oxoglutarate
Proline
Fructose
Myo−inositol
PC2
PC1
C
8 WPE
PC2 (22.1%)
PC1 (30.3%)
Asparagine
Quinic_acid
Glucose
Proline
Aspartate
Alanine
Fructose
Galactose
Uridine
Arginine
Cytidine
Choline
Tyrosine
4−Aminobutyrate
Phenylalanine
Lysine
Leucine
Threonine
Valine
Isoleucine
PC2
PC1
D
12 WPE
PC2 (22.9%)
PC1 (30.2%)
Glucose
Quinic_acid
Fructose
Myo−inositol
Galactose
Alanine
Choline
Aspartate
Proline
Phenylalanine
Leucine
Threonine
Sucrose
2−Oxoglutarate
Lysine
Isoleucine
Arginine
Valine
Asparagine
4−Aminobutyrate
PC2
PC1

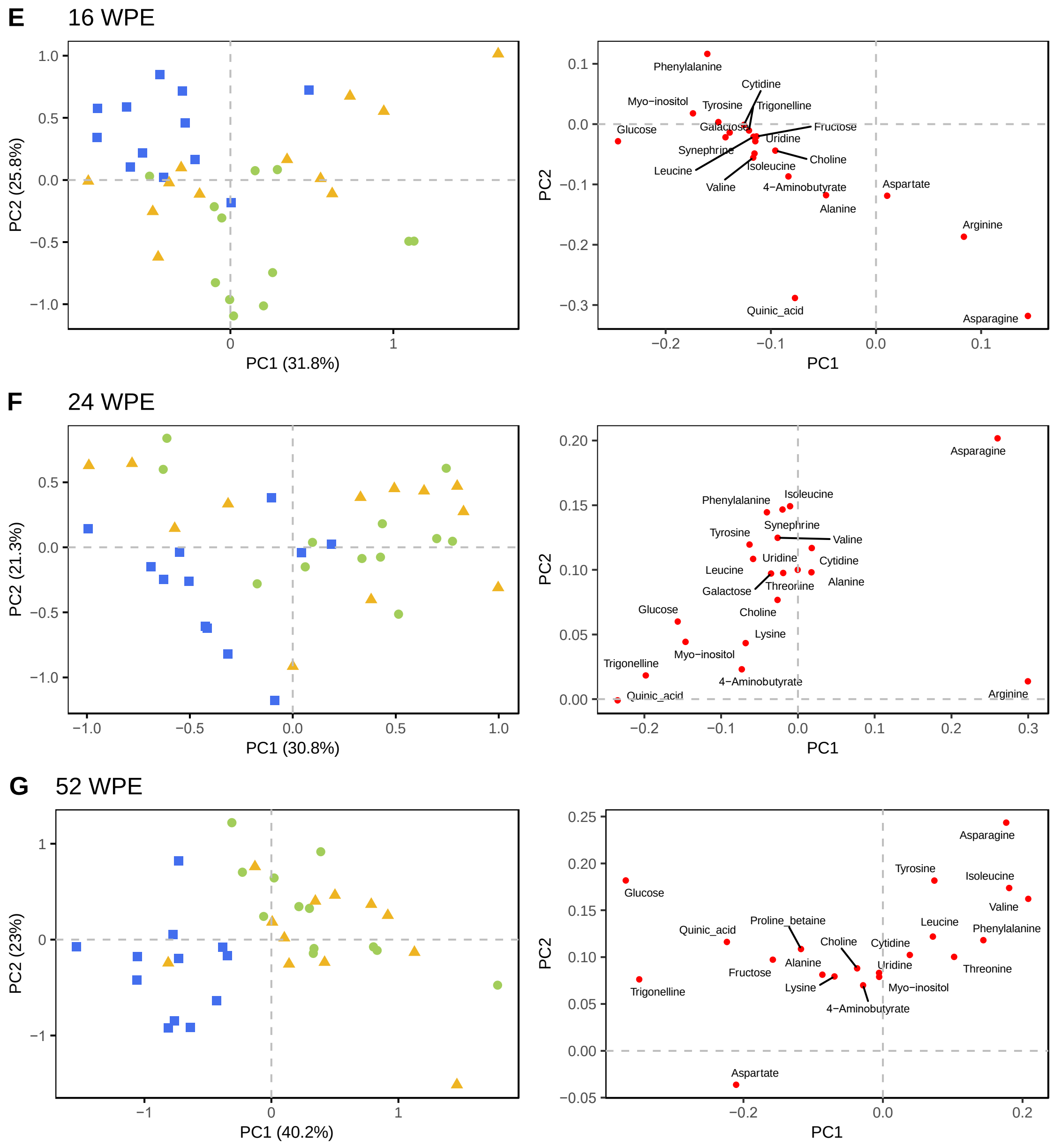

Figure S4. Metabolomics principal component analysis (PCA) and corresponding loadings plot showing the top 20 variables contributing to principal component 1 (PC1) and principal component 2 (PC2) for 0, 4, 8, 12, 16, 24, and 52 wpe (A-G).

References:

(1) Chin, E.; Godfrey, K.; Polek, M.; Slupsky, C. ^{1}H NMR analysis of *Citrus macrophylla* subjected to Asian citrus psyllid (*Diaphorina citri* Kuwayama) feeding. *Arthropod-Plant Interact* **2017**, *11* (6), 901-909. DOI: 10.1007/s11829-017-9546-0.

(2) Chin, E. L.; Ramsey, J. S.; Mishchuk, D. O.; Saha, S.; Foster, E.; Chavez, J. D.; Howe, K.; Zhong, X.; Polek, M.; Godfrey, K. E.; et al. Longitudinal transcriptomic, proteomic, and metabolomic analyses of *Citrus sinensis* (L.) Osbeck graft-inoculated with " *Candidatus* Liberibacter asiaticus". *J Proteome Res* **2020**, *19* (2), 719-732. DOI: 10.1021/acs.jproteome.9b00616.
